# Morphological and Molecular Diversity of Phytoplankton in Beibu Gulf, Northern South China Sea

**DOI:** 10.1002/ece3.71207

**Published:** 2025-04-09

**Authors:** Shalini Thevarajan, Pengfei Sun, Pengbin Wang, Jie Xu, Jie Chen, Yongyu Tan, Junjie Zheng, Mengmeng Tong

**Affiliations:** ^1^ Ocean College Zhejiang University Zhoushan China; ^2^ Key Laboratory of Tropical Marine Ecosystem and Bioresource, Fourth Institute of Oceanography Ministry of Natural Resources Beihai China; ^3^ Guangxi Beibu Gulf Key Laboratory of Marine Resources, Environment and Sustainable Development, Fourth Institute of Oceanography Ministry of Natural Resources Beihai China; ^4^ Key Laboratory of Marine Ecosystem Dynamics, Second Institute of Oceanography Ministry of Natural Resources Hangzhou China; ^5^ Centre for Regional Oceans & Department of Ocean Science and Technology, Faculty of Science and Technology University of Macau Macau China

**Keywords:** 18S rDNA, Beibu gulf, environmental driver, metabarcoding, morphology, phytoplankton

## Abstract

The Beibu Gulf, a vital region for marine biodiversity and aquaculture, is increasingly affected by nutrient‐driven ecological shifts in the phytoplankton community. This study combined morphology and eDNA metabarcoding (18S rDNA V4) to investigate phytoplankton diversity and environmental drivers during summer and winter in the Beibu Gulf. Metabarcoding detected 3.5 times more phytoplankton species, contributing to higher species diversity and richness than morphology. Metabarcoding identified 200 phytoplankton genera from eight phyla, while morphology only identified 49 genera from six phyla. Both methods revealed different dominant phytoplankton communities. Bacillariophyta and Haptophyta dominated the phytoplankton community based on morphology, in summer and winter, respectively; meanwhile, Dinophyta dominated in both seasons under metabarcoding due to their high 18S rDNA copy number. Altogether, 83 HAB and/or toxic species were identified, among which 10 were dominant, suggesting a high risk of HAB outbreaks in the Beibu Gulf. Phytoplankton abundance increased from south to north and west to east in both seasons, following the high input of dissolved inorganic nitrogen (DIN) and silicate. Excess ammonium input can promote the dominance of *Scrippsiella trochoidea* and *Heterocapsa circularisquama*, positioning them as emerging HAB species, while excess DIN caused extreme phosphorus limitation and favored the dominance of *Phaeocystis globosa* in the Beibu Gulf. This study provided a comprehensive description of the influence of environmental drivers on the phytoplankton community in the Beibu Gulf.

## Graphical abstract

This study explored phytoplankton diversity and environmental drivers in Beibu Gulf based on morphology and metabarcoding. The findings reveal that morphology better reflected phytoplankton abundance while metabarcoding revealed the actual phytoplankton diversity. Excess anthropogenic nitrogen input caused severe P‐limitation and favored the dominance of *Phaeocystis globosa* in Beibu Gulf.

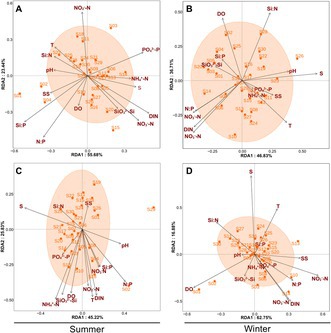

## 1 Introduction

Beibu Gulf (17°–22° N, 105.5°–110° E) is a semi‐enclosed bay shared by Vietnam and China, situated in the northwest of the South China Sea and comprises a total area of approximately 130,000 km^2^. It is an economically important mariculture base and a major fishing ground in China because it harbors significant marine ecosystems with steady marine production (Gao et al. 2017). The biological productivity in the gulf is ascribable to the stable nutrient supply from three main water masses, namely, the coastal current, South China Sea water, and West‐Guangdong coastal current. The coastal current and South China Sea water are the dominant contributors of nutrients in summer and winter, respectively. The West‐Guangdong coastal current originates from the west coast of Guangdong Province and flows into the gulf through Qiongzhou Strait, is stable throughout the year (Chen et al. 2019; Lao et al. 2022). Following the recent development in economy and urbanization, these water masses carry a great amount of nutrient load from the agricultural, domestic, and industrial waste originating from the coastal cities of Guangdong Province, Hainan Island, and Guangxi Province into the gulf (Lao et al. 2021; Zhu et al. 2022). As a result, the coastal waters of Qinzhou Bay, Fangcheng Bay, Lianzhou Bay, and Dafeng Estuary, situated at the northern Beibu Gulf, had become eutrophic, leading to the expansion of Harmful Algal Blooms (HABs) caused by *Microcystis* spp., *Trichodesmium erythraeum* , *Phaeocystis globosa* , *Skeletonema costatum* , *Guinardia flaccida* , *Leptocylindrus danicus* , and *Noctiluca scintillans* (Lai et al. 2014; Xu et al. 2019; Wang 2022). Anthropogenic activities directly influence the distribution and abundance of phytoplankton due to their fast lifespan and sensitivity to environmental changes (Ponnusamy et al. 2023).

Although extensive data has been gathered on phytoplankton in Beibu Gulf since 1999, much of these data was obtained based on morphological identification (Boonyapiwat 1999; Zhuang et al. 2011; Lai et al. 2014; Qin et al. 2023). The morphological technique, including light microscopy, is reliable to be used on species with apparent and unique morphological features; however, it is difficult to be applied to small‐sized and cryptic phytoplankton species (Huo et al. 2020). In recent years, DNA‐based methods have been developed and effectively used alone or in combination with microscopy to classify phytoplankton (Tzafesta et al. 2022). Environmental DNA (eDNA) metabarcoding based on 18S rDNA has efficiently identified phytoplankton present in a natural environment (Huo et al. 2020; He et al. 2023). The V4 region of 18S rDNA is the most variable region with a highly conserved primer binding site, which is well suited for biodiversity assessments because of its ability to detect a wide range of phylogenetically distant taxa (Marinchel et al. 2023). eDNA metabarcoding with the 18S rDNA V4 marker has successfully identified HAB species in the Gulf of Mexico, Yellow Sea, and the West Pacific (Liu et al. 2020; Huang et al. 2021; Gaonkar and Campbell 2023). In Beibu Gulf, this molecular marker has successfully identified the HAB and pico‐phytoplankton genera such as *Amoebophyra*, *Karlodinium*, *Prorocentrum*, *Gymnodinium*, *Ostreococcus*, and *Micromonas* (He et al. 2023; Wang, Gu, et al. 2024). It can distinguish most species of dinoflagellates and certain diatom species (e.g., *Nitzschia* spp. and *Gomphonema* spp.) however, it could possibly miss the detection of certain haptophytes, such as *Prymnesium parvum* and *Isochrysis galbana* , green algae, and heterotrophs (Visco et al. 2015; Stuart et al. 2024).

Therefore, it is essential to combine both identification methods to determine the actual phytoplankton diversity and composition in Beibu Gulf. In this study, morphology and eDNA metabarcoding (based on 18S rDNA V4) methods were applied to determine the diversity, abundance, and distribution of phytoplankton in Beibu Gulf. Previous studies conducted in this area focused mainly on either one, morphology or molecular identification technique, which may not provide an accurate composition of the phytoplankton species (Boonyapiwat 1999; Zhuang et al. 2011; Lai et al. 2014; He et al. 2023; Wang, Gu, et al. 2024). Moreover, most studies focused on a small‐scale area, which does not represent the entire Beibu Gulf (Zhuang et al. 2011; Ge et al. 2023; He et al. 2023; Wang, Schneider, et al. 2024). Therefore, to determine the morphological and molecular phytoplankton diversity and better understand the environmental influence on the dominance of the phytoplankton species in Beibu Gulf, two cruises were conducted in the summer and winter of 2022 to: (1) explain the spatiotemporal distribution and the potential environmental driver(s) for the phytoplankton dominance determined based on morphology and metabarcoding techniques and (2) elucidate the strengths and limitations of morphology and molecular approaches for phytoplankton identification.

## 2 Materials and Methods

### 2.1 Study Area

**FIGURE 1:**
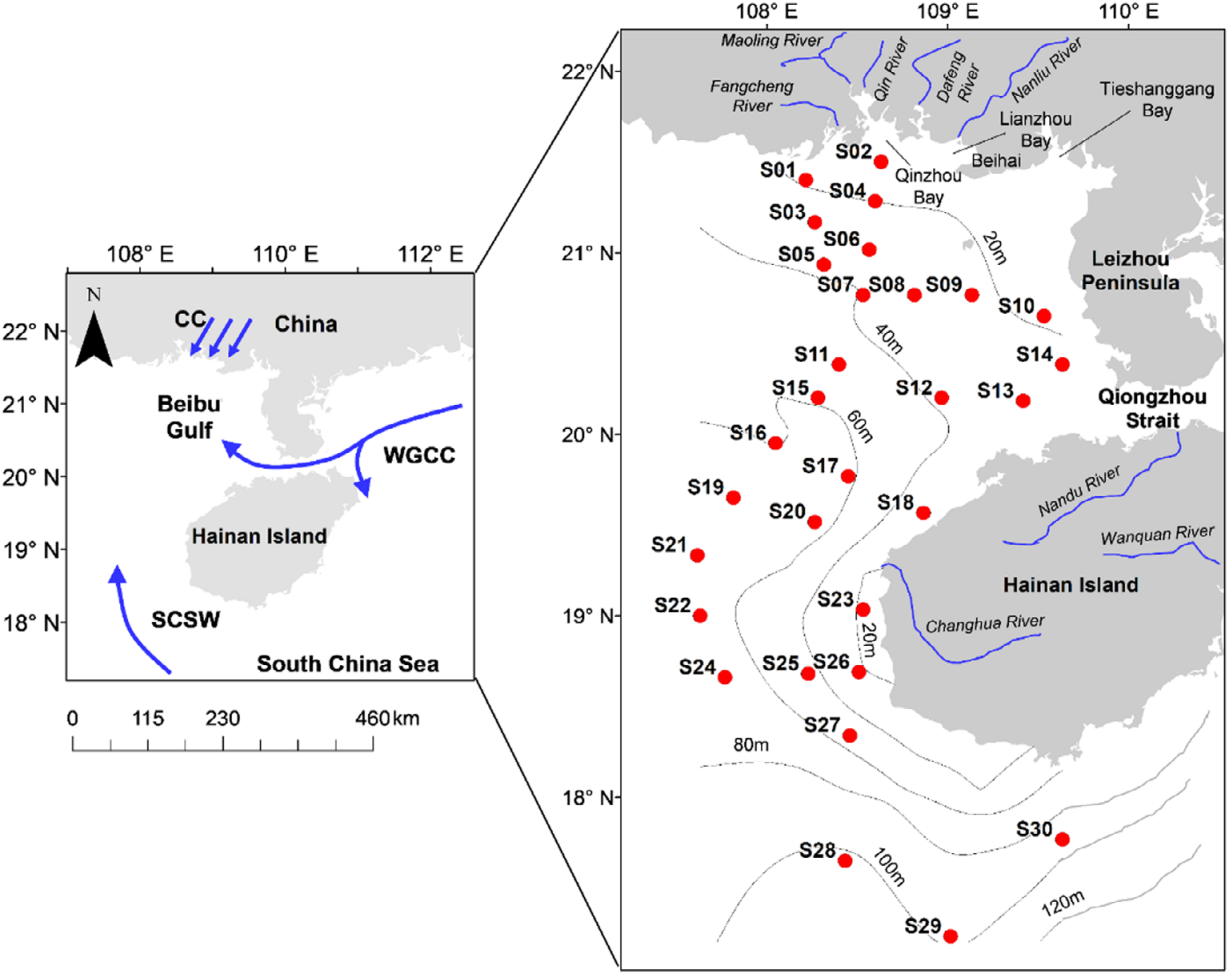
The study area and sampling stations (red dots) in Beibu Gulf, China. The blue arrows in the left figure represents the main water masses in the gulf, modified from Lao et al. (2022). CC, coastal current from Guangxi Province; SCSW, South China Sea water; WGCC, West‐Guangdong coastal current.

The Beibu Gulf (17°–22° N, 105.5°–110° E) covers an area of 130,000 km^2^ with a 1629 km coastline (Xu et al. 2019). Two cruises were conducted in the west of Beibu Gulf from 12 August 2022 to 31 August 2022 (summer) and 11 December 2022 to 11 January 2023 (winter). In total, 30 sampling locations were selected for physical, chemical, and biological analyses (S01–S30) (Figure 1). The two seasons were chosen to represent the seasonal extremes in the study area (Nie et al. 2012).

### 2.2 Nutrient and Water Quality Analysis

The in situ measurement of sea surface temperature (T), sea surface salinity (S), pH, and dissolved oxygen (DO) was taken using a YSI multi‐parameter water quality analyzer (YSI INC, USA). Meanwhile, a conductivity‐temperature‐depth profiler (CTD) was used to measure the depth of each sampling station (RBR Concerto, Canada). Replicates of 1 L surface water samples were collected for seawater physical and chemical analyses at each station each cruise. The collected samples were filtered on‐board using Whatman GF/F filters (47 mm diameter, 0.42 mm thick, 0.7 μm pore size) under low vacuum pressure. The filters and filtrate were stored at −20°C for chlorophyll *a* (Chl‐*a*) and nutrient analyses, respectively.

Chl‐*a* was detected following the method proposed by Su et al. (2010). Filters frozen at −20°C were immersed in 10 mL of 90% acetone in the dark for 24 h to extract Chl‐*a*. Then, they were centrifuged at 3500 *g* for 10 min to obtain a clear extraction. Chl‐*a* content was measured using a spectrophotometer (U‐2910 Hitachi) at 630, 647, 664, and 750 nm. Silicate (${\mathrm{SiO}}_{3}^{2-}–\mathrm{Si}$), phosphate (${\mathrm{PO}}_{4}^{3-}–P$), ammonium (${\mathrm{NH}}_{4}^{+}–N$), nitrate (${\mathrm{NO}}_{3}^{-}–N$), nitrite (${\mathrm{NO}}_{2}^{-}–N$) and dissolved inorganic nitrogen (DIN) were chosen for chemical analysis because they are the most relevant macronutrients necessary for phytoplankton growth and are primarily available in the inorganic forms (Barcelos e Ramos et al. 2017). Flow injection analysis (QuikChem 8500) was done to quantify the concentration of ${\mathrm{SiO}}_{3}^{2-}–\mathrm{Si}$, ${\mathrm{PO}}_{4}^{3-}–P$, ${\mathrm{NH}}_{4}^{+}–N$, ${\mathrm{NO}}_{3}^{-}–N$, and ${\mathrm{NO}}_{2}^{-}–N$ (Knap et al. 1994; Pai et al. 2001; Han et al. 2012). DIN was measured by the addition of ${\mathrm{NH}}_{4}^{+}–N$, ${\mathrm{NO}}_{3}^{-}–N$, and ${\mathrm{NO}}_{2}^{-}–N$. Suspended solids were measured by filtering 1 L of seawater through a pre‐weighed glass fiber filter, drying it at 100°C, and weighing it again.

### 2.3 Species Identification

A 1.5 L of surface and bottom water samples were collected using a Ruttner sampler and transferred into a clean polyethene bottle. The samples were fixed immediately with 2% Lugol's iodine solution. Then the samples were homogenized and transferred to a 10 mL Hydro‐bios counting chamber in which they were let to settle for 48 h (Utermohl 1958). Before enumeration, the lower layer containing the sedimented phytoplankton was covered using a cover slip while the upper layer was decanted. Phytoplankton enumeration was carried out in triplicates by examining at 40× and 100× magnification using a microscope (Olympus CKX53). The abundance of phytoplankton was calculated as cells per liter (cells L^−1^) regardless of size (LeGresley and McDermott 2012). The phytoplankton were identified up to the species or genus level based on morphological characteristics and standard references (Cupp 1943; Desikachary 1959; Hendy 1964; Hallegraeff et al. 2004).

### 2.4 DNA Extraction and Sequencing

For molecular‐based identification, 100 to 200 mL aliquots from surface and bottom water samples of each station were filtered with 0.22 μm filters and stored at −80°C until next generation sequencing (NGS) analysis. Prior to NGS analysis, DNA from each sample was extracted following the manual instructions of the MicroElute Genomic DNA Kit, Omega. The purity and quantity of extracted DNA were assessed with a NanoDrop spectrophotometer. Then, polymerase chain reaction (PCR) amplification was carried out on the V4 region of the 18S rDNA using the primers: 528F (5′‐GCGGTAATTCCAGCTCCAA‐3′) and 706R (5′‐AATCCRAGAATTTCACCTCT‐3′) (He et al. 2023). The PCR reaction was performed with a final volume of 30 μL containing 1 μL of forward primer, 1 μL of reverse primer, 15 μL of PCR Phusion Master Mix with GE Buffer (Biolabs) mix, and 1 ng of DNA template. The PCR reaction conditions were set as follows: initial denaturation at 94°C for 1 min, followed by 30 cycles of denaturation at 94°C for 1 min, annealing at 58°C for 30 s, and elongation at 72°C for 2 min, with a final extension at 72°C for 3 min. PCR products were then sent for Illumina HiSeq sequencing at Shanghai Personalbio Biotechnology Co. Ltd., China.

### 2.5 Sequence Data Analysis

The quality of raw reads obtained from Illumina HiSeq sequencing was checked using fastQC and filtered by removing primers and chimera sequences using DADA2 to obtain high quality reads. The filtered reads were clustered into operational taxonomic units (OTUs) using VSEARCH based on 97% sequence similarity. The abundance statistics of OTUs were obtained and only OTUs with an abundance value of more than one were retained. Taxonomic annotation was performed using the NCBI database for each OTU representative sequence obtained and the relative abundance of each OTU was calculated using the flattening process in QIIME2 (2019.4).

### 2.6 Statistical Analysis

The phytoplankton and OTU distribution map was created using ArcMap (version 10.7.1, available at https://www.esri.com/en‐us/arcgis/geospatial‐platform/overview) to visualize the spatial distribution pattern of phytoplankton identified. The gradient map for spatial distribution of physical and chemical parameters was plotted using Ocean Data View version 4.7 (Schlitzer 2019). Two‐tailed independent‐sample *t*‐tests with Levene's test for equality of variance were performed using SPSS software version 25.0 to compare the statistical significance of seasonal variations in physical and chemical parameters, phytoplankton abundance, OTU reads, and diversity indexes. For further statistical analyses, both phytoplankton cell and OTU abundance, as well as nutrient concentrations, were log transformed using the formula log(*x* + 1). Bray–Curtis dissimilarity index (Bray and Curtis 1957) was performed in R Package version 4.3.1 (R Core Team 2020) on the transformed data, and Non‐metric Multidimensional Scaling (NMDS) was used to visualize the results. The computed Bray–Curtis dissimilarity index was used to perform UPGMA‐based hierarchical clustering to compare the similarities in the distribution of the dominant phytoplankton identified in both seasons.

Further, Redundancy Analysis (RDA) was performed on the log(*x* + 1) transformed data in R Package version 4.3.1 (R Core Team 2020) to compute the relationship between environmental variables and dominant phytoplankton taxa. Prior to performing RDA, Detrended Correspondence Analysis (DCA) was conducted using the “decorana” function in R to assess the appropriacy of RDA for the data. The DCA analysis yielded an axis length of < 4, indicating RDA was suitable for the data. Following that, Spearman Correlation analysis and Mantel's test were performed between the dominant phytoplankton taxa and alpha diversity indexes with the environmental variables, in R using the “cor” function and “mantel” function. The species richness was estimated by calculating Margalef Index (*D*) (Donald 1990) following the formula:

$$D=\left(S-1\right)/\log_{2}N,$$

and the species diversity was estimated using the Shannon‐Wiener index (H') (Shannon 1948), following the formula:

$$H^{'}=-\sum_{i=1}^{S}P_{i}\times \log_{2}P_{i},$$

Pielou's index (J') (Pielou 1969) was used to estimate the evenness of phytoplankton according to the formula:

$$J^{'}=\left(H^{'}\right)/\left(\log_{2}S\right),$$

The dominance index (*Y*) proposed by (Mcnaughton 1967) was used to determine the dominant species from both seasons and was calculated using:

$$Y=n_{i}/N\times f_{i}$$

In the equation: *S* = number of identified species in a sample; *P*_*i*_ = relative species biomass of the ith species; *n*_*i*_ = number of cells/read number of the ith morphospecies/OTU; *N* = total abundance/read number of cells/OTU and *f*_*i*_ = occurrence frequency of the *i*th morphospecies/OTU.

## 3 Results

### 3.1 Physiochemical Parameters

The sea surface temperature (*T*) and pH in summer ranged from 28.3°C to 32.0°C and 8.16 to 8.51, were significantly (*p* < 0.01) higher than winter with a range of 16.2°C to 25.9°C and 7.93 to 8.27, respectively. In contrast, the surface salinity (S), dissolved oxygen (DO), Chl‐*a*, nitrate (${\mathrm{NO}}_{3}^{-}–N$), nitrite (${\mathrm{NO}}_{2}^{-}–N$), DIN, and phosphate (${\mathrm{PO}}_{4}^{3-}–P$) in summer ranged from 24.9 to 33.0 psu, 5.6 to 8.6 mg L^−1^, 0.10 to 2.42 μg L^−1^, 0.02 to 6.21 μmol L^−1^, 0.01 to 1.06 μmol L^−1^, 0.33 to 9.92 μmol L^−1^, and 3.23 × 10^3^ to 0.46 μmol L^−1^, were significantly (*p* < 0.05) lower than winter with a range of 30.7 to 34.0 psu, 6.7 to 8.1 mg L^−1^, 0.91 to 6.80 μg L^−1^, 0.01 to 25.71 μmol L^−1^, 0.07 to 1.89 μmol L^−1^, 0.87 to 29.49 μmol L^−1^, and 0.02 to 1.25 μmol L^−1^, respectively. The seasonal difference in suspended solids (SS), ammonium (${\mathrm{NH}}_{4}^{+}–N$), and silicate (${\mathrm{SiO}}_{3}^{2-}–\mathrm{Si}$) was not significant (*p* > 0.05) with a range of 0.1 to 33.1 mg L^−1^, 0.04 to 11.94 μmol L^−1^, and 7.65 to 104.38 μmol L^−1^, respectively (Tables S1 and S2).

The distribution of T, S, DO, and Chl‐*a* showed great spatiotemporal variation. T followed an increasing trend from south to north in summer, while the opposite was observed in winter. S increased from north to south, and the opposite was recorded for DO and Chl‐*a* in both seasons. The highest T and pH were recorded at the Guangxi coast and the southern gulf in summer and winter, respectively. The Guangxi coast also recorded the lowest S, meanwhile the opposite was observed for DO in both seasons. SS was concentrated at the west of Leizhou Peninsula and Hainan Island. Additionally, high Chl‐*a* concentration was recorded at the west of Leizhou Peninsula in summer and the Guangxi coast in winter (Figure S1).

The nutrients, ${\mathrm{NO}}_{3}^{-}–N$, DIN, ${\mathrm{NO}}_{2}^{-}–N$, and ${\mathrm{SiO}}_{3}^{2-}–\mathrm{Si}$ were found to be concentrated mainly at the west coast of Leizhou Peninsula and Guangxi coast (Figure S2). The distribution of ${\mathrm{NO}}_{3}^{-}–N$, ${\mathrm{NH}}_{4}^{+}–N$, DIN, and ${\mathrm{SiO}}_{3}^{2-}–\mathrm{Si}$ followed a decreasing trend from north to south and east to west in both seasons. ${\mathrm{NO}}_{2}^{-}–N$ in summer was highly concentrated in the northwest coast of Hainan Island, meanwhile, in winter, it followed an increasing pattern from west to east. In summer, ${\mathrm{PO}}_{4}^{3-}–P$ was concentrated at the northeast, meanwhile, in winter, it followed an increasing trend from east to west. If the Redfield ratio (Redfield 1960) is considered for the stoichiometry of these elements, Beibu Gulf was generally phosphorus limited (P‐limited), with an average N:Si:P ratio of 77:1250:1 and 96:337:1 in summer and winter, respectively. The distribution of the N:P ratio follows a decreasing trend from north to south and east to west (Figure S2).

### 3.2 Phytoplankton Identification

#### 3.2.1 Morphological Identification of Phytoplankton

The phytoplankton abundance in summer and winter ranged from 3.18 × 10^5^ to 2.34 × 10^8^ and 9.62 × 10^4^ to 8.19 × 10^7^ cells L^−1^, respectively. Average phytoplankton abundance in summer (4.33 × 10^7^ cells L^−1^) was significantly (*p* = 0.007) higher than in winter (7.66 × 10^6^ cells L^−1^). The morphological method identified 90 (summer) and 84 (winter) morphospecies, classified within six Phyla: Bacillariophyta, Chlorophyta, Cyanophyta, Haptophyta, Ochrophyta, and Dinophyta. Among classes, Coscinodiscophyceae (66.26%), Fragilariophyceae (24.11%), Trebouxiophyceae (7.19%), Mediophyceae (1.18%), Bacillariophyceae (0.75%), Cyanophyceae (0.26%), and Dinophyceae (0.18%) were the main classes found in summer. In winter, Prymnesiophyceae (63.28%) became dominant, followed by Coscinodiscophyceae (16.58%), Fragilariophyceae (12.30%), Mediophyceae (3.31%), Bacillariophyceae (2.25%), Cyanophyceae (1.96%), and Dinophyceae (0.10%).

**FIGURE 2:**
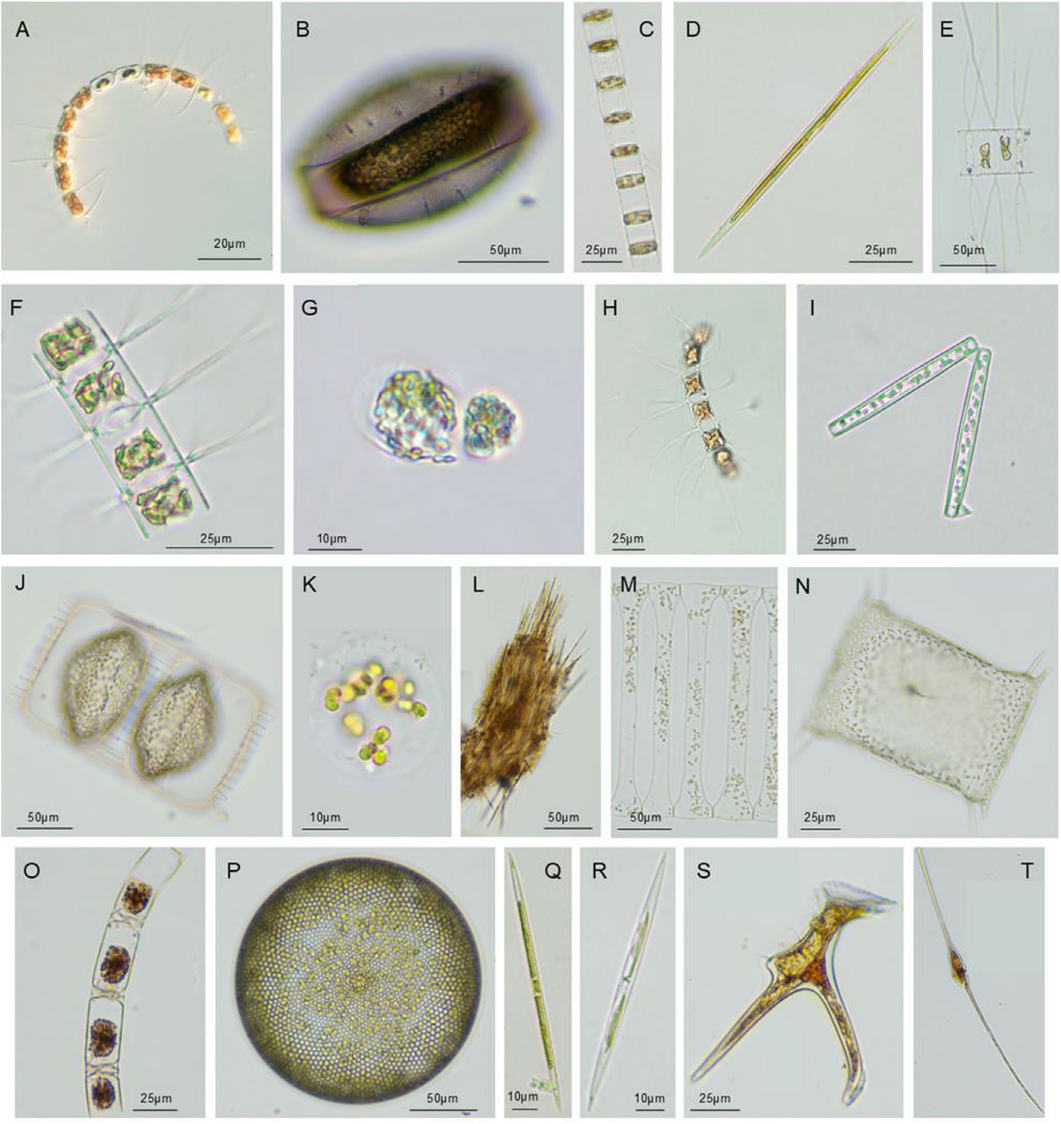
The top 10 most abundant and potentially toxic phytoplankton morphospecies identified in summer and winter (A–T). (A) *Chaetoceros curvisetus* ; (B) *Thalassiosira subtilis* ; (C) *Skeletonema costatum* ; (D) *Synedra* spp.; (E) *Chaetoceros decipiens* ; (F) *Bacteriatrum hyalinum*; (G) *Chorella* spp.; (H) *Chaetoceros pseudocurvisetus* ; (I) *Thalassionema nitzschioides* ; (J) *Cyclotella* spp.; (K) *Phaeocystis globosa* ; (L) *Trichodesmium erythraeum* ; (M) *Climacodium frauenfeldianum* ; (N) *Odontella sinensis* ; (O) *Bellerochea horologicalis* ; (P) *Coscinodiscus asteromphalus* ; (Q) *Pseudo‐nitzschia delicatissima* ; (R) *Pseudo‐nitzschia pungens* ; (S) *Dinophysis miles*; (T) *Ceratium fusus* .

**FIGURE 3:**
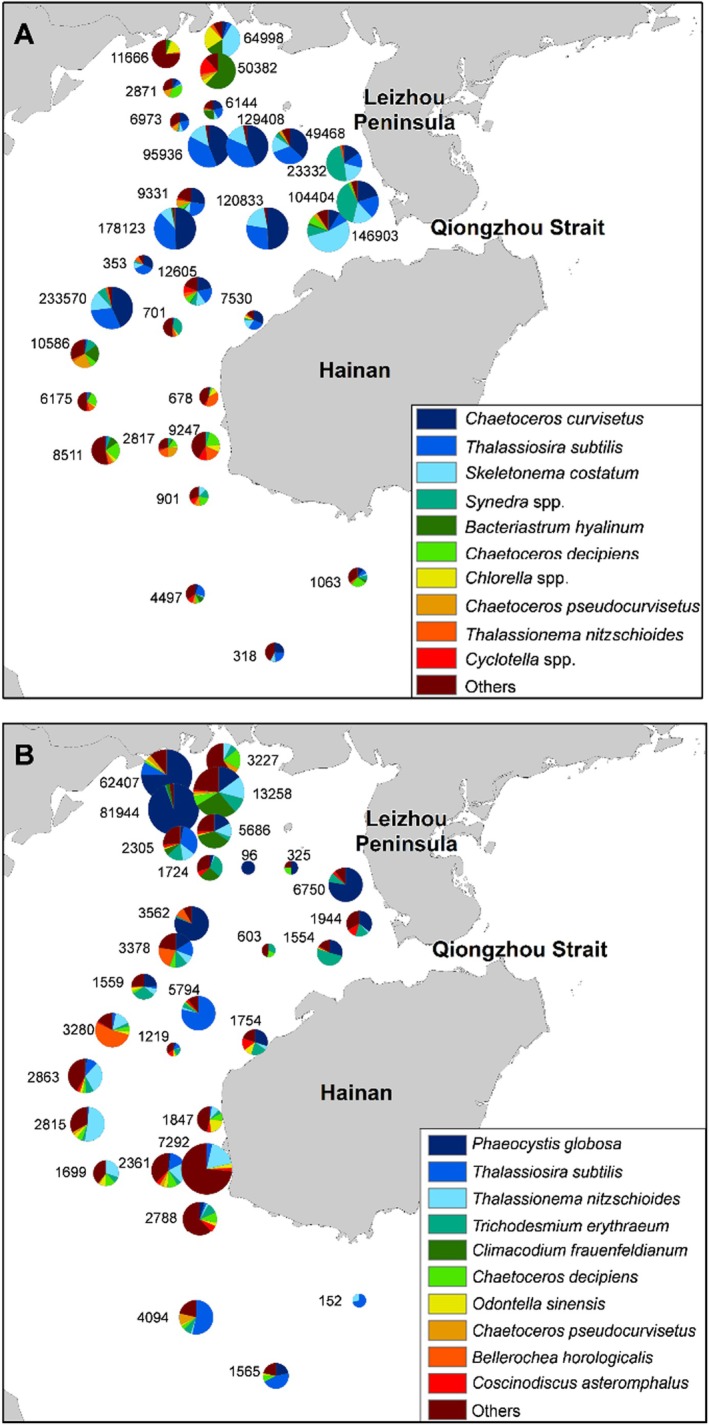
The spatial distribution of phytoplankton and the composition of the top 10 most abundant phytoplankton taxa in: (A) Summer and (B) winter determined by a morphological approach. Each pie chart is labeled with the corresponding phytoplankton cell abundance (×10^3^ cells L^−1^) of the sampling station.

In summer, members of Bacillariophyta, *Chaetoceros curvisetus* , *Thalassiosira subtilis* , *Skeletonema costatum* , *Synedra* spp. (Figure 2A–D), and *Bacteriastrum hyalinum* (Figure 2F) dominated the phytoplankton community (Table S3), exhibiting clear abundance in the northern coastal areas (Figure 3A). Among 90 morphospecies identified, in summer, 39 were HAB species, whereby four ( *Pseudo‐nitzschia delicatissima* , *Trichodesmium erythraeum* , *Ceratium furca*, and *Dinophysis miles*) were potentially toxic (Table S4). In winter, Haptophyta became dominant, with *Phaeocystis globosa* (Figure 2K) being the most abundant species, occupying mainly the northern coastal areas. Additionally, the diatoms *Thalassiosira subtilis* , *Thalassionema nitzschioides* (Figure 2I), and cyanobacteria, *T. erythraeum* (Figure 2L) were also dominant in winter and distributed mainly from the west to south of Hainan Island (Figure 3B). Among 84 morphospecies identified, 33 were HAB species, whereby four ( *Pseudo‐nitzschia pungens* , *T. erythraeum* , *P. globosa*, and *D. miles*) were potentially toxic.

#### 3.2.2 Metabarcoding Identification of Phytoplankton

The total OTU reads in summer and winter ranged from 4.88 × 10^4^ to 1.60 × 10^5^ OTUs and 3.96 × 10^4^ to 1.72 × 10^5^ OTUs, respectively. Average OTU reads in winter (1.00 × 10^5^ OTU) were significantly higher (*p* = 0.025) than summer (8.38 × 10^4^ OTU). In total, 5622 and 5281 different OTU sequences were obtained and annotated into 359 and 373 phytoplankton taxa in summer and winter, respectively, based on 97% sequence similarity. The phytoplankton taxa were classified into eight Phyla: Dinophyta, Chlorophyta, Bacillariophyta, Haptophyta, Rhodophyta, Streptophyta, Ochrophyta, and Cryptophyta. The major phytoplankton taxa in summer were comprised of Dinophyceae (71.01%), Mamiellophyceae (10.06%), Coscinodiscophyceae (5.00%), Chloropicophyceae (2.95%) and Trebouxiophyceae (2.05%). In winter, the major phytoplankton taxa were comprised of Dinophyceae (64.36%), Mamiellophyceae (9.78%), Coscinodiscophyceae (7.46%), Cryptophyceae (3.39%), Trebouxiophyceae (3.30%), and Chloropicophyceae (2.82%).

**FIGURE 4:**
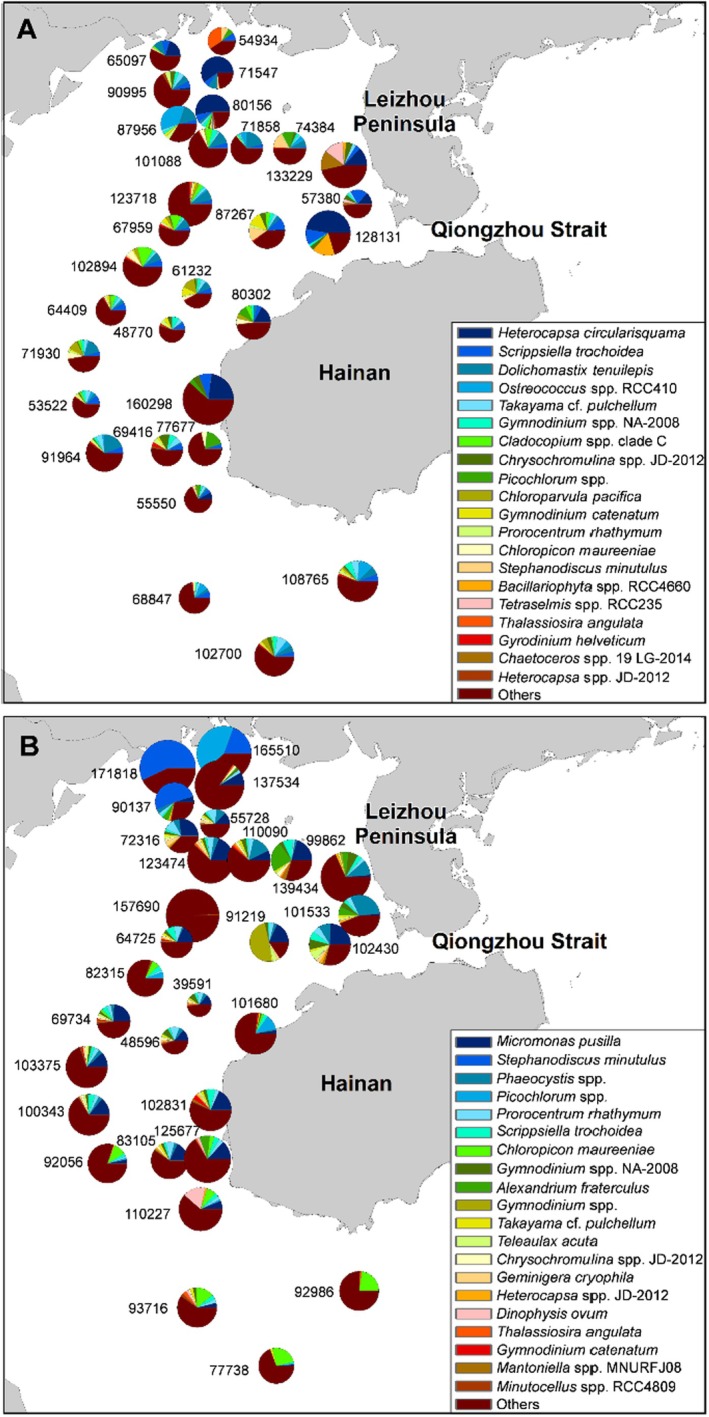
The spatial distribution of OTU and the composition of the top 20 most abundant phytoplankton taxa in: (A) Summer and (B) winter determined by the metabarcoding approach. Each pie chart is labeled with the corresponding total OTU read number of the sampling station.

According to metabarcoding, Dinophyta and Chlorophyta dominated the phytoplankton community in both seasons. In summer, *Heterocapsa circularisquama*, *Scrippsiella trochoidea* , *Dolichomastix tenuilepis*, and *Ostreococcus* spp. RCC410, *Takayama* cf. *pulchellum*, and *Gymnodinium* spp. NA‐2008 were the most dominant taxa (Table S3) and were highly abundant in the north and east coastal areas (Figure 4A). Among the 359 taxa detected in summer, 48 were HAB species, with 15 being potentially toxic (Table S5). In winter, the pico‐phytoplankton *Micromonas pusilla* was the most dominant taxon and was distributed throughout the gulf; meanwhile, *Stephanodiscus minutulus* , *Phaeocystis* spp., *Picochlorum* spp., *Prorocentrum rhathymum*, *S. trochoidea* , and *Chloropicon maureeniae* were also dominant while occupying the northeast coastal areas (Figure 4B). Among the 373 taxa detected in winter, 48 were HAB species, with 16 being potentially toxic.

#### 3.2.3 Changes in Dominant Phytoplankton Composition in Beibu Gulf

**FIGURE 5:**
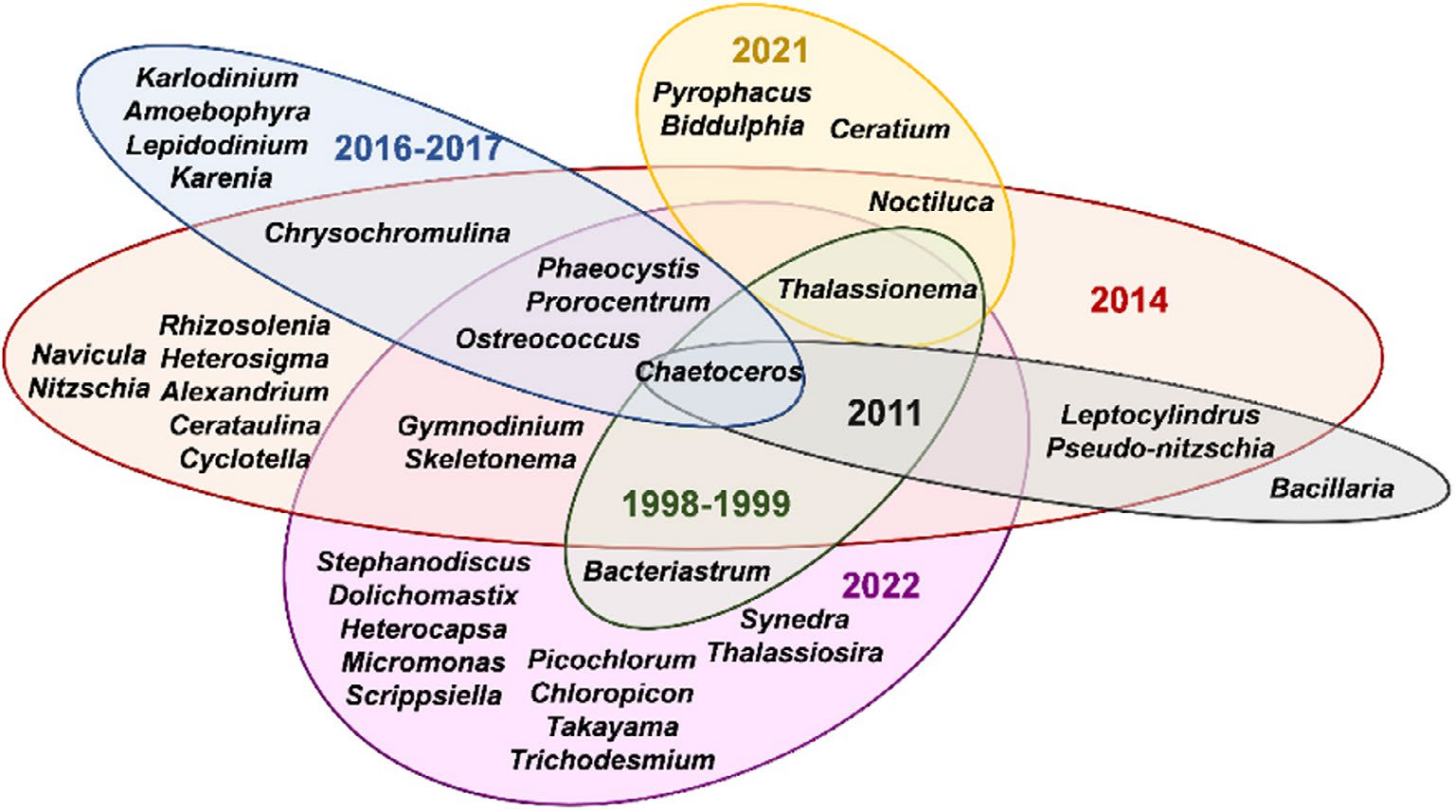
Dominant phytoplankton genera recorded at Beibu Gulf in previous studies conducted in the years 1998 to 2022. Reference: 1998–1999 (Cai et al. 2008), 2011 (Fujing et al. 2015), 2014 (Lai et al. 2017), 2016–2017 (He et al. 2023), 2021 (Huiling et al. 2023) and 2022 (This study).

Figure 5 illustrates the dominant phytoplankton genera in Beibu Gulf over the years. Before 2014, the phytoplankton community in Beibu Gulf was largely dominated by diatoms from genera *Chaetoceros*, *Bacteriastrum*, and *Thalassionema*. The HAB‐causing haptophyte, *Phaeocystis*, has been consistently dominating after the year 2011. From the year 2014, the HAB‐causing dinoflagellates from genera *Prorocentrum*, *Alexandrium*, *Noctiluca*, and *Gymnodinium* began dominating the phytoplankton community. Ever since, the dominance and diversity of harmful dinoflagellates identified in Beibu Gulf have been increasing. Meanwhile, the dominant harmful dinoflagellates from genera *Heterocapsa*, *Scrippsiella*, and *Takayama*, in this study, have not been dominant before in Beibu Gulf, indicating the potential for emerging HAB species. These findings highlight the increasing dominance of HAB species over the years and the need for consistent monitoring of phytoplankton composition in Beibu Gulf.

### 3.3 Comparison of the Two Identification Methods

**FIGURE 6:**
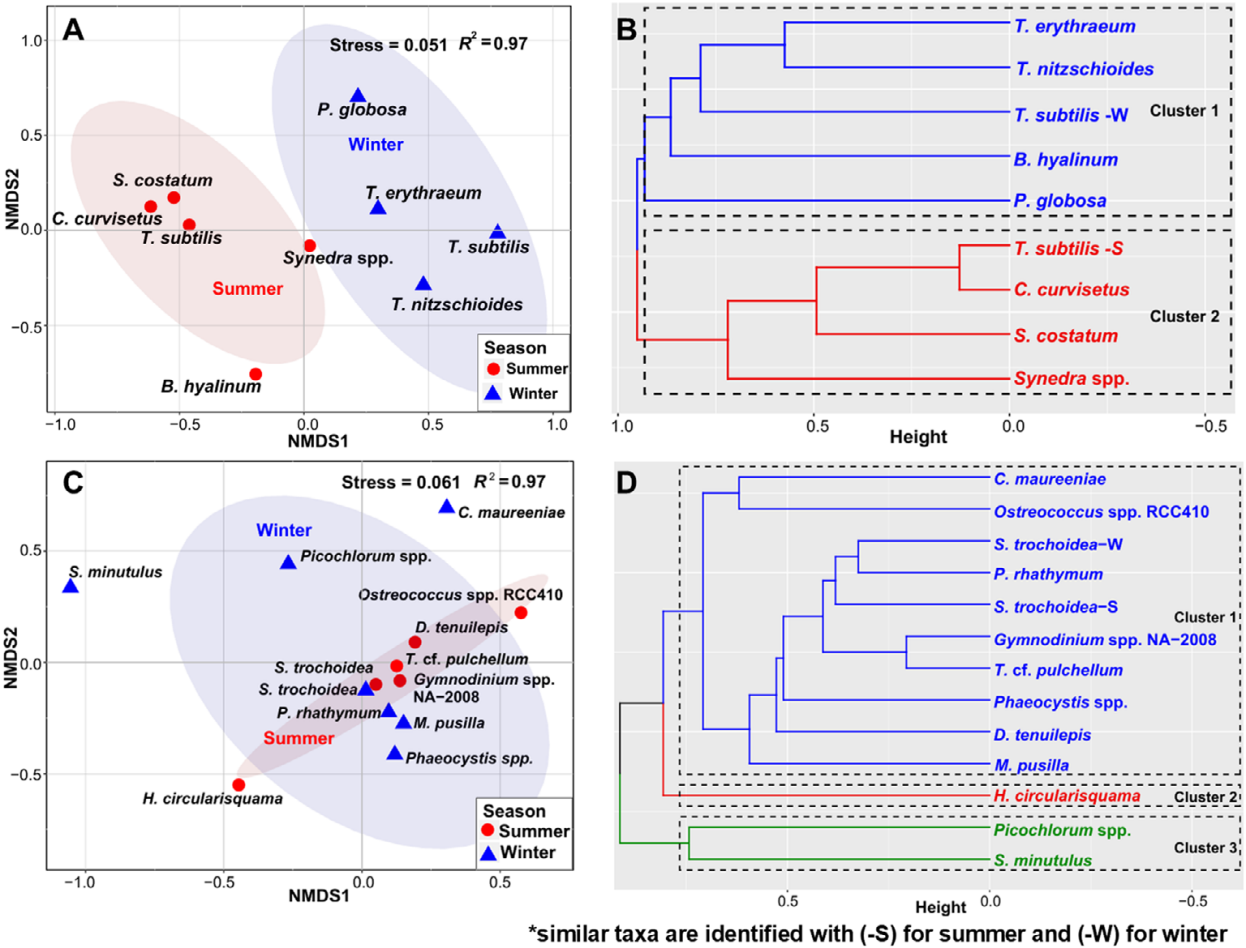
Non‐multidimensional scaling (NMDS) based on Bray–Curtis dissimilarity index and UPGMA‐based hierarchical clustering of the dominant phytoplankton. (A, B) represents the dominant morphospecies identified in the morphological approach; (C, D) represents the dominant taxa identified in the metabarcoding approach.

Based on the dominance index, 20 dominant phytoplankton taxa (*Y >* 0.02) were identified (Table S3) among which 10 were HAB species. The Bray–Curtis Dissimilarity Index was calculated between the dominant phytoplankton taxa. Based on morphological identification, members of Bacillariophyta demonstrated higher similarity in the distribution in summer; meanwhile, *Thalassiosira subtilis* demonstrated high seasonal variation (Figure 6A). The UPGMA‐based hierarchical clustering separated the dominant taxa from both seasons into two different clusters, indicating clear seasonal variation (Figure 6B). Based on metabarcoding analysis, *Heterocapsa circularisquama* demonstrated high dissimilarity in distribution with the other dinoflagellates (Figure 6C) and the hierarchical clustering demonstrated a closer relationship between *Phaeocystis* spp. and the dinoflagellates (Figure 6D).

**FIGURE 7:**
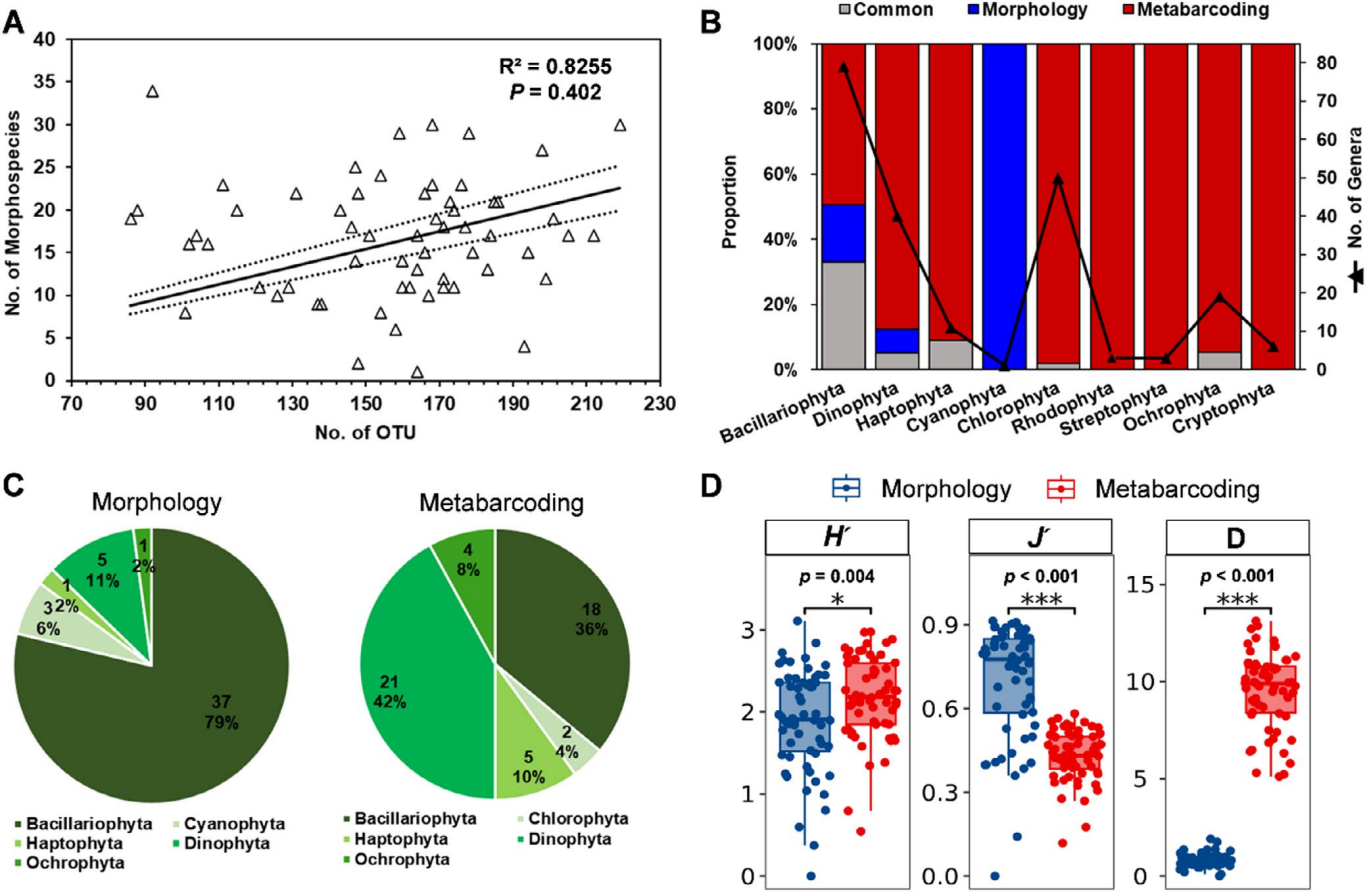
The (A), linear regression analysis between the number of OTU and morphospecies identified (B), proportion of genera commonly detected or solely found with the morphological and metabarcoding approach (C), percentage of HAB species identified by both methods and (D), average Shannon‐Wiener Index (H′), Pielou's Index (J′), and Margalef Index (D) compared between morphology and metabarcoding.

A significantly high number of phytoplankton OTUs (86–218) were identified compared to the number of morphospecies (1–34) (*p* < 0.01) however, these measures were not significantly correlated (*R* ^2^ = 0.8255, *p* = 0.402) (Figure 7A). The phytoplankton composition identified by both methods was compared at the generic level due to the differences in taxonomic resolution and the need for consistency in comparison. Both methods detected 31 genera in common; meanwhile, 18 and 168 genera were solely detected by morphological and metabarcoding methods, respectively (Table S6). Metabarcoding was able to detect more phytoplankton genera from Chlorophyta (49 Genera), Dinophyta (35 genera), Ochrophyta (18 genera) and Haptophyta (10 genera) however, Cyanophyta was not detected (Figure 7B). Morphology detected 47 HAB species, while metabarcoding identified 50 HAB species. Both methods identified 14 HAB species in common, whereby 12 belonged to Bacillariophyta (Figure 7C). The seasonal differences between alpha diversity indexes were insignificant (*p* > 0.05) (Table S7). Both the Shannon‐Wiener (*p* = 0.004) and Margalef (*p* < 0.001) indexes in metabarcoding were significantly higher than morphology; meanwhile, Pielou's index in morphology was significantly (*p* < 0.001) higher than metabarcoding (Figure 7D).

### 3.4 Relationship of the Dominant Phytoplankton Taxa With the Environmental Variables

**FIGURE 8:**
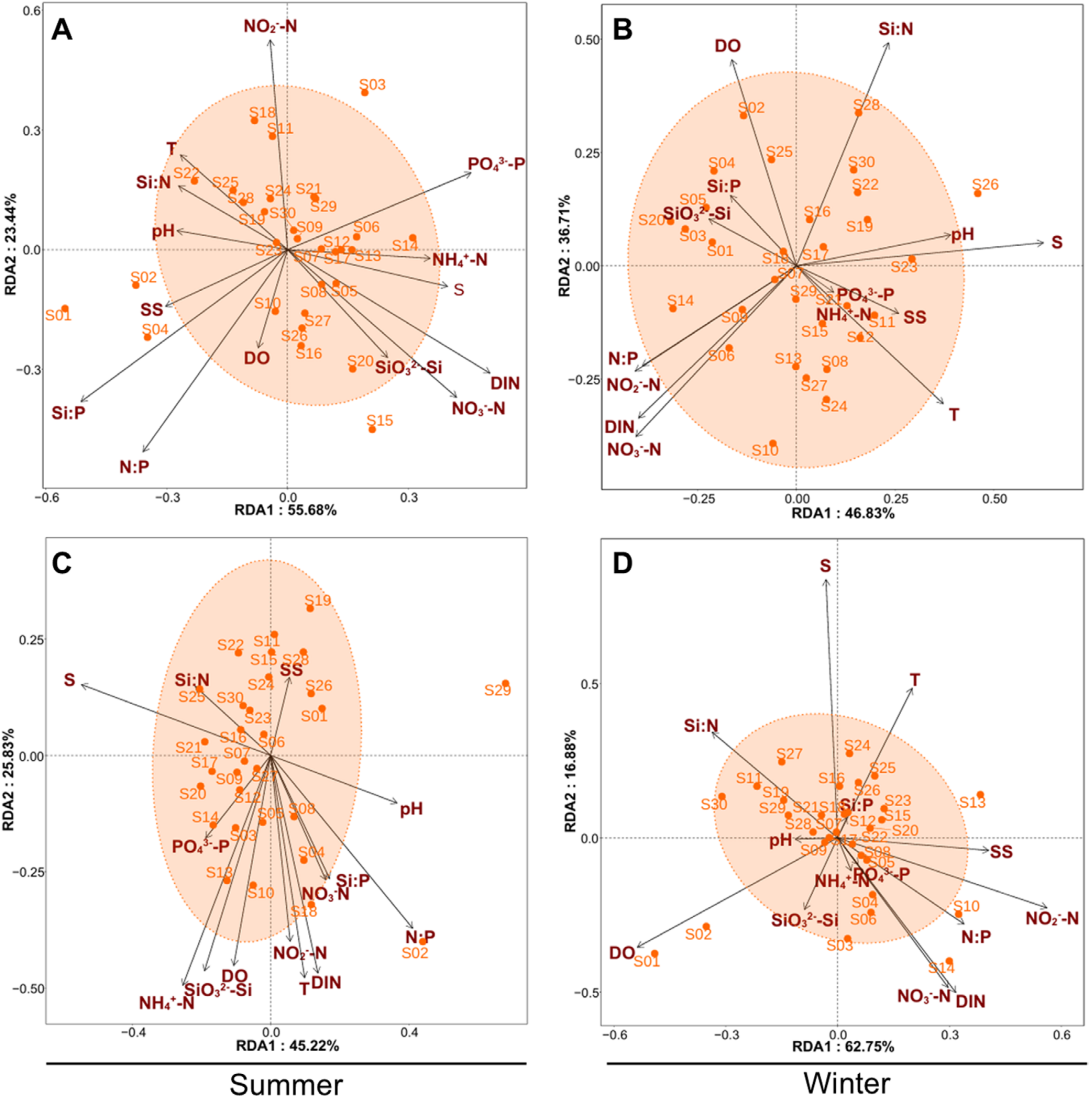
Ordination plot of redundancy analysis (RDA) describing the relationship between phytoplankton community with temperature (T), salinity (S), dissolved oxygen (DO), pH, suspended solids (SS), ${\mathrm{NO}}_{3}^{-}–N$, ${\mathrm{NO}}_{2}^{-}–N$, ${\mathrm{NH}}_{4}^{+}–N$, DIN, ${\mathrm{SiO}}_{3}^{2-}–\mathrm{Si}$, ${\mathrm{PO}}_{4}^{3-}–P$, N:P, Si:N, and Si:P. Note: (A) and (B) Note: RDA based on morphological identification in summer and winter, respectively (C) and (D). RDA based on metabarcoding identification in summer and winter, respectively.

**FIGURE 9:**
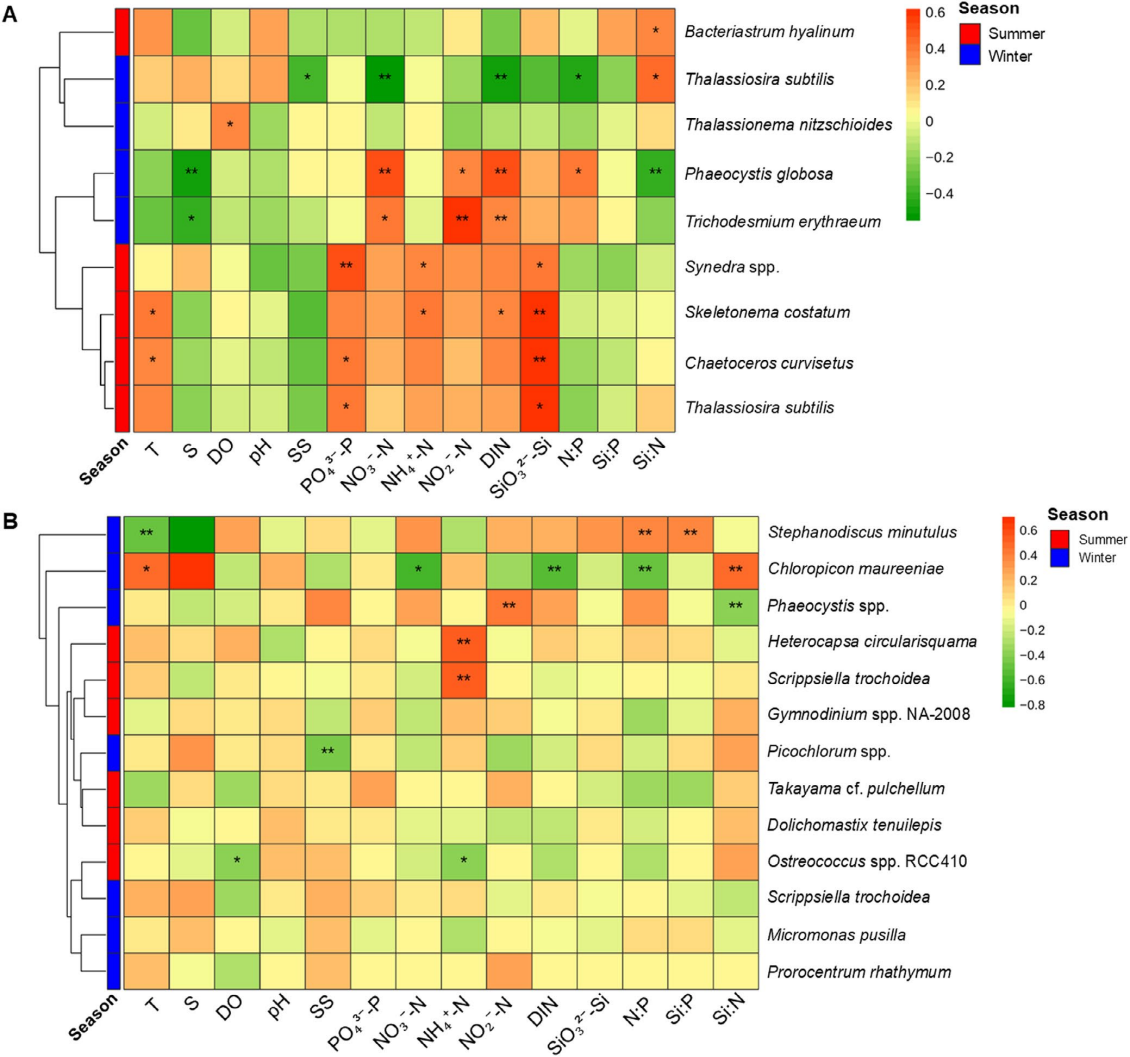
Spearman correlation analysis between the dominant phytoplankton taxa identified in (A) morphology and (B) metabarcoding with temperature (T), salinity (S), dissolved oxygen (DO), pH, suspended solids (SS), ${\mathrm{NO}}_{3}^{-}–N$, ${\mathrm{NO}}_{2}^{-}–N$, ${\mathrm{NH}}_{4}^{+}–N$, DIN, ${\mathrm{SiO}}_{3}^{2-}–\mathrm{Si}$, ${\mathrm{PO}}_{4}^{3-}–P$, N:P, Si:N and Si:P. The ‘*’ represents *p* < 0.05 and ‘**’ represents *p* < 0.01.

The RDA was conducted between environmental variables and the phytoplankton community. Based on morphology, the RDA explained 79.12% (Figure 8A) and 83.54% (Figure 8B) of the variation in the phytoplankton community in summer and winter, respectively, and the phytoplankton community was mainly affected by S, N:P, and Si:P ratios (*p* < 0.05). Based on metabarcoding, the RDA explained 70.52% (Figure 8C) and 79.55% (Figure 8D) of the variation in the phytoplankton community in summer and winter, respectively, and the phytoplankton community was mainly affected by S, DO, DIN, ${\mathrm{NO}}_{3}^{-}–N$, and ${\mathrm{NO}}_{2}^{-}–N$. Spearman correlation analysis was performed between the environmental variables and the dominant phytoplankton taxa. Based on morphology, in summer, ${\mathrm{SiO}}_{3}^{2-}–\mathrm{Si}$ positively correlated to the diatoms, *Skeletonema costatum* , *Chaetoceros curvisetus* (*p* < 0.01) and *Thalassiosira subtilis* (*p* < 0.05). In winter, DIN and ${\mathrm{NO}}_{3}^{-}–N$ positively correlated with *Phaeocystis globosa* (*p* < 0.01) and *Trichodesmium erythraeum* (*p* < 0.05) and negatively correlated with *T. subtilis* (*p* < 0.01) (Figure 9A). Based on metabarcoding, in summer, ${\mathrm{NH}}_{4}^{+}–N$ positively correlated with the harmful dinoflagellates, *Heterocapsa circularisquama* and *Scrippsiella trochoidea* (*p* < 0.01). In winter, T positively correlated with *Stephanodiscus minutulus* (*p* < 0.01) and negatively correlated with *Chloropicon maureeniae* (*p* < 0.05) (Figure 9B). Mantel's test was used to explore the relationship between phytoplankton alpha diversity and environmental factors. Based on morphology, Shannon–Wiener and Pielou's indexes were significantly correlated with T (*p* < 0.01), while the Margalef index was significantly correlated with ${\mathrm{NO}}_{3}^{-}–N$ (*p* < 0.05) in summer. However, none of the alpha diversity indexes in metabarcoding demonstrated significant correlations with the environmental factors (Figure S3).

## 4 Discussion

### 4.1 Environmental Influence on Phytoplankton Composition

In the present study, a marked increase of phytoplankton abundance was observed from south to north and west to east, in both seasons correlating with the nutrient distribution, mainly DIN and ${\mathrm{SiO}}_{3}^{2-}–\mathrm{Si}$ (Figure S2). The northern coast of the gulf receives anthropogenic nutrient inputs from agriculture, aquaculture, marine industries, and port areas delivered by the Qiongzhou Strait and tributary rivers, resulting in the accumulation of excess DIN, ${\mathrm{SiO}}_{3}^{2-}–\mathrm{Si}$ and ${\mathrm{PO}}_{4}^{3-}–P$ at the Guangxi coast and west of Leizhou Peninsula (Wang et al. 2018; Lao et al. 2021; Lu et al. 2022; Zhang et al. 2023). Additionally, large quantities of ${\mathrm{PO}}_{4}^{3-}–P$ were also contributed by the Red River flowing through heavy agricultural and urban activity areas in Vietnam, which discharges at the west of the gulf (Hoang et al. 2017). Excess nutrient input from Qiongzhou Strait and tributary rivers likely contributed to the rapid proliferation of opportunistic species from Bacillariophyta, Dinophyta, and Haptophyta at the northern coastal areas, favoring the increase in phytoplankton abundance. Previous studies have shown that in coastal areas with high nutrient inputs, the phytoplankton communities often shift toward fast‐growing species, leading to increased phytoplankton biomass (Bužančić et al. 2016; Taipale et al. 2019). Most areas, especially at the northern region of the gulf, were P‐limited (Pan et al. 2021), with the limitation intensifying in winter (Figure S2). This could also alter the phytoplankton community by favoring species that can tolerate low phosphorus conditions such as *Phaeocystis globosa* (Wang, Song, et al. 2021). The phenomenon of P‐limitation leading to a shift in community composition has been observed before in the South Pacific Ocean and West Atlantic coast (Trommer et al. 2013; Liu et al. 2024).

Based on morphology, Bacillariophyta dominated the phytoplankton composition in summer, which shifted to Haptophyta in winter. He et al. (2023) reported that Bacillariophyta were the main phytoplankton group identified under microscopic observation during summer in Beibu Gulf. In this study, Sperman correlation revealed a positive correlation between ${\mathrm{SiO}}_{3}^{2-}–\mathrm{Si}$ and the dominant members from Bacillariophyta, *Chaetoceros curvisetus* , *Thalassiosira subtilis* , *Skeletonema costatum* , and *Synedra* spp. (Figure 9A) which were abundant on the west coast of Leizhou Peninsula (Figure 3A). This suggests that excess ${\mathrm{SiO}}_{3}^{2-}–\mathrm{Si}$ contributed by anthropogenic activities facilitated the proliferation of these diatoms under suitable temperature (Pan et al. 2023). However, the overall diatom abundance reduced in winter compared to summer and shifted to being dominated by the haptophyte, *Phaeocystis globosa* (Figure 3B). *P. globosa* has caused recurring HABs in Beibu Gulf during winter since 2011 (Niu et al. 2022). A previous study mentioned that *P. globosa* in Beibu Gulf usually blooms after the diatom bloom due to the availability of ${\mathrm{SiO}}_{3}^{2-}–\mathrm{Si}$ (Wang, Song, et al. 2021). In this study, the summer ${\mathrm{SiO}}_{3}^{2-}–\mathrm{Si}$ concentration was higher than winter (Table S2). The reduction in the winter ${\mathrm{SiO}}_{3}^{2-}–\mathrm{Si}$ concentration may have allowed *P. globosa* to outcompete the diatoms, leading to a shift in phytoplankton composition. Additionally, *P. globosa* was positively correlated with ${\mathrm{NO}}_{3}^{-}–N$ (Figure 9A). Similarly, Zhu et al. (2023) reported that the *P. globosa* bloom occurred in Beibu Gulf when the ${\mathrm{NO}}_{3}^{-}–N$ concentration was three times more than ${\mathrm{NH}}_{4}^{+}–N$. Moreover, *P. globosa* is known to form HABs in phosphorus‐limited conditions (Wang, Song, et al. 2021; Qin et al. 2023; Zhou et al. 2025), which could elevate the possibility of this species dominating the phytoplankton community of Beibu Gulf, since it is largely a P‐limited ecosystem (Zhu et al. 2023). Therefore, excessive ${\mathrm{NO}}_{3}^{-}–N$ input and P‐limited conditions in Guangxi coast and west of Leizhou Peninsula contributed to the dominance of *P. globosa* in these areas under suitable temperature.

According to metabarcoding, Dinophyta was the most abundant group in both seasons; however, the abundance in summer was higher than in winter. Meanwhile, both Dinophyta and Chlorophyta co‐dominated the phytoplankton community composition in winter. Conducive temperatures in summer promoted the growth of harmful dinoflagellates like *Heterocapsa circularisquama* and *Scrippsiella trochoidea* , covering the northern coastal areas (Figure 4A), as both these species prefer higher temperatures for optimum growth (Yamaguchi et al. 1997; Tian et al. 2021). The HAB of *H. circularisquama*, reported in western Japanese waters and Kuwait Bay, caused mass shellfish mortalities (Matsuyama 2012; Saburova et al. 2022). Furthermore, the resting cysts of *S. trochoidea* have been detected in the South China Sea and are highly abundant throughout the year (Tang et al. 2021). Moreover, a positive association of ${\mathrm{NH}}_{4}^{+}–N$ with both *H. circularisquama* and *S. trochoidea* was revealed in Spearman correlation (Figure 9B). *H. circularisquama* shows a higher uptake preference for ${\mathrm{NH}}_{4}^{+}–N$ over ${\mathrm{NO}}_{3}^{-}–N$, likely to reduce energy expenditure for nitrogen assimilation, while ${\mathrm{NH}}_{4}^{+}–N$ availability is known to increase cyst formation in *S. trochoidea* (Wang et al. 2014; Yamamoto et al. 2017). In recent decades, a significant elevation in ${\mathrm{NH}}_{4}^{+}–N$ input into the Chinese coastal waters has been reported due to the expansion of aquaculture activities (Wang, Liu, et al. 2021). The continuous ${\mathrm{NH}}_{4}^{+}–N$ input may favor the dominance of *H. circularisquama* and *S. trochoidea* in Beibu Gulf, increasing the risk of HABs and shifting the dominant phytoplankton composition. Meanwhile, in winter, the pico‐phytoplankton *Micromonas pusilla* was the most dominant taxon (Figure 4B), contributing largely to the abundance of Chlorophyta. The reduction in temperature during winter could have allowed *M. pusilla* to outcompete the dominant dinoflagellates because it is well adapted to lower temperatures and reportedly has high abundance among the pico‐phytoplankton communities in Beibu Gulf; however, microscopic observation could miss its detection due to its small size (Lovejoy et al. 2007; Lin et al. 2021).

### 4.2 Strengths and Limitations of Morphology and Molecular Approaches for Phytoplankton Identification

In this study, about 15% of the phytoplankton genera were commonly reported in both methods, indicating that both identification techniques provide a reasonably comparable detection of the easily recognizable phytoplankton taxa. Metabarcoding, with high sequencing depth, identified 3.5 times more phytoplankton taxa, contributing to higher species diversity and richness, meanwhile lower species evenness compared to morphology. Metabarcoding in this study detected the rare members of the phytoplankton community, Rhodellophyceae, Klebsormidiophyceae, Bolidophyceae, and Raphidophyceae, contributing to the high phytoplankton diversity. A similar result was obtained by Šimunović et al. (2023) for the phytoplankton community in Lake Visovac. On the contrary, Wang et al. (2022) reported a higher species diversity, richness, and evenness of metabarcoding over morphological identification in the South China Sea. This discrepancy in species evenness could be due to the clear dominance of members from Dinophyceae over the diverse phytoplankton taxa detected in metabarcoding. Moreover, metabarcoding excelled in detecting the small pico‐phytoplankton genera (*Bathycoccus*, *Ostreococcus*, *Micromonas*, *Prasinococcus*, *Nannochloris*, *Picocystis*, *Pycnococcus*, *Minidiscus*, *Imantonia*) (Table S6), adding to the species diversity and richness compared to morphology. Despite their vast diversity and abundance, pico‐phytoplankton in the Beibu Gulf remain poorly documented, highlighting the potential for future studies to benefit from advanced techniques such as metabarcoding.

However, some limitations of metabarcoding need to be addressed. The choice of gene marker is essential in obtaining the highest resolution in metabarcoding results. The 18S rDNA V4 region is a reliable choice for eDNA metabarcoding of eukaryotic phytoplankton community (Huo et al. 2020; Liu et al. 2020; He et al. 2023). In this study, the 18S rDNA V4 marker was effective for detecting members of Chlorophyta (44 species) and Dinophyta (41 species), which were missed in morphology; however, it might not be suitable to detect the members of Cyanophyta or some diatoms due to the possibility of primer bias. The 18S rDNA V4 marker was sufficient to distinguish the genus *Chlorella* up to species level (*C. stigmatophora*, *C. vulgaris* and *C. sorokiniana* ), proving its efficiency for identifying green algae (Zou et al. 2018) when morphology failed to distinguish them. Nevertheless, this gene marker was insufficient to identify genus *Trichodesmium* and *Bacteriastrum* belonging to Cyanophyta and Bacillariophyta, respectively. Prokaryotes like Cyanobacteria are better targeted using the 16S rDNA or ITS regions (MacKeigan et al. 2022) because of the absence of 18S rDNA in them. Even so, 16S rDNA is unsuitable for detecting eukaryotic phytoplankton because they acquire chloroplasts through diverse endosymbiosis origins (Kim and Archibald 2010). Therefore, the high‐resolution areas such as 16s rDNA (MacKeigan et al. 2022) or ITS (Ballesteros et al. 2021) can be used in metabarcoding studies to specifically target members of Cyanophyta. Further, the presence of spliceosomal introns identified in the 18S rDNA V4 region of diatoms from genera *Chaetoceros* and *Bacteriastrum* could disrupt the primer binding sites and interrupt sequencing; hence, they can be missed in metabarcoding (Gaonkar et al. 2018, 2020). Instead, *rbcL* and *cox1* gene markers might be better candidates for targeting diatoms. Turk Dermastia et al. (2023) claimed *rbcL* detected more diatom genera, including 22 new species in the Adriatic Sea, compared to 18S. Meanwhile, *cox1* could provide species‐level distinction of diatoms; however, the lack of a universal primer for this marker might hinder its application in metabarcoding (Evans et al. 2007; Moniz and Kaczmarska 2009).

It is certainly controversial to analyze and correlate 18S rDNA sequences with cell abundance following the large copy number variation in 18S rDNA among phytoplankton species (Zhu et al. 2005). In this study, Dinophyta dominated the OTU read numbers comprising more than 60% in both seasons, while in morphology, Bacillariophyta and Haptophyta dominated the cell numbers in summer and winter, respectively, leading to vast variation in the dominant phytoplankton community identified by both methods. The 18S rDNA copy number varies substantially among phytoplankton and largely correlates to species size (Zhu et al. 2005). The 18S rDNA genomic copy number in diatoms and dinoflagellates could reach up to 166 and 4919 copies cell^−1^, respectively (Martin et al. 2022), causing an overestimation of dinoflagellate OTU read numbers which has been widely reported (Liu et al. 2020; Wang et al. 2022), while leading to the underestimation of the dominant diatoms, *Skeletonema costatum* , *Chaetoceros curvisetus* , and *Synedra* spp. in the metabarcoding dataset. This proves that the morphological approach provides a much more reliable cell count that reflects the actual phytoplankton abundance in an environment; meanwhile, metabarcoding is more useful in determining the actual phytoplankton diversity (Huo et al. 2020).

Among the 418 OTUs identified in this study, only 206 OTUs (49.28%) were identified at the species level, and 167 OTUs (39.95%) at the genus level after being annotated against the NCBI database. Several genera (14 diatoms and three dinoflagellates) and species (71 diatoms and 16 dinoflagellates) were detected only using morphology, highlighting the critical gap in the reference database. Some of these genera (*Detonmula*, *Gossleriella*, *Climacodium*, *Pyrocystis* and *Pyrophacus*) were absent or lacked 18S reference sequences in the database. In this case, using a regularly curated taxonomic database like SILVA, designed particularly for ribosomal RNA sequences, could offer a much better species‐level resolution (Wang, Schneider, et al. 2024). He et al. (2023) identified 41 and 37 species of dinoflagellates and diatoms, respectively, in Beibu Gulf using SILVA, indicating that this database provides equal resolution for both these phytoplankton groups. Additionally, the diat.barcode, a curated database designated specifically for diatoms, has also proven to be effective for the 18S V4 marker (Bailet et al. 2020). This study used a 97% similarity threshold for OTU clustering, which is widely accepted for microbial studies; however, it could underestimate the phytoplankton diversity and restrict species‐level detection (Mysara et al. 2017; Rzehak et al. 2024). Previous studies reported that many closely related microalgal and fungal species can share nearly identical 18S rDNA sequences with > 98% sequence similarity (Tedersoo et al. 2022; Bjorbaekmo et al. 2023). Therefore, taxonomists often consider a more stringent (e.g., 99%) similarity threshold to be suitable for distinguishing species (Garnica et al. 2016; Rzehak et al. 2024). Notably, even organisms that share a high level of sequence similarity may not represent the same species; hence, the possibilities of underestimating phytoplankton diversity using 18S rDNA may be unavoidable (Fox et al. 1992; Mysara et al. 2017).

## 5 Conclusions

This study used both the morphology and eDNA metabarcoding (18S rDNA V4) to determine the phytoplankton diversity and distribution in Beibu Gulf. Metabarcoding detected 3.5 times more phytoplankton taxa, contributing to higher species diversity and richness compared to morphology. The spatial distribution of phytoplankton abundance increased from south to north and west to east in both seasons, following the excess anthropogenic nutrient input in these areas. Metabarcoding and morphology inferred different dominant phytoplankton communities. According to morphology, Bacillariophyta was the most abundant group in summer, which then shifted to Haptophyta in winter. Meanwhile, Dinophyta dominated the phytoplankton community in both seasons, according to metabarcoding. Excess ${\mathrm{NH}}_{4}^{+}–N$ input led to the dominance of *S. trochoidea* and *H*. *circularisquama* in summer, positioning them as emerging HAB species. Further, continuous nitrogen input could lead to extreme P‐limitation and favor the HAB outbreak of *P. globosa* , making this species a potential indicator for anthropogenic eutrophication in Beibu Gulf. While morphology better reflected the phytoplankton abundance, the sensitivity of metabarcoding revealed the actual phytoplankton diversity. Nevertheless, the integration of morphology and metabarcoding revealed the actual phytoplankton community composition and HAB diversity in Beibu Gulf.

## Author Contributions

**Shalini Thevarajan:** data curation (equal), formal analysis (equal), software (equal), visualization (equal), writing – original draft (equal), writing – review and editing (equal). **Pengfei Sun:** conceptualization (equal), funding acquisition (equal), project administration (equal), resources (equal), supervision (equal). **Pengbin Wang:** conceptualization (equal), supervision (equal), validation (equal). **Jie Xu:** resources (equal), visualization (equal), writing – review and editing (equal). **Jie Chen:** conceptualization (equal), resources (equal), validation (equal). **Yongyu Tan:** investigation (equal), software (equal), visualization (equal). **Junjie Zheng:** methodology (equal), resources (equal). **Mengmeng Tong:** conceptualization (equal), supervision (equal), validation (equal).

## Ethics Statement

The animal study was reviewed and approved by the Fourth Institute of Oceanography, Ministry of Natural Resources, and Zhejiang University.

## Conflicts of Interest

The authors declare no conflicts of interest.

## Supporting information


Data S1.

## Data Availability

The raw sequences are publicly available on NCBI under the BioProject ID “PRJNA1155855.”
