# Supplementary material for: Morphological and Molecular Diversity of Phytoplankton in Beibu Gulf, Northern South China Sea
Source: Ecol Evol. 2025 Apr 9;15(4):e71207. doi: 10.1002/ece3.71207 (PMC11981879; doi:10.1002/ece3.71207)
Supplement: Supplementary file 1 — Data S1. [file ECE3-15-e71207-s001.zip › ece371207-sup-0002-supinfo02..docx]

# **Supplementary Materials**

## **Seasonal Variations of Abiotic Factors**

Table 1: Two-tailed independent-sample t-tests between the mean value of abiotic factors (mean ± SD) in summer and winter in Beibu Gulf, China.

| **Physical Parameters** | **Unit** | **Summer** | **Winter** | ***p*** |
| --- | --- | --- | --- | --- |
| Temperature (T) | ℃ | 30.6±0.7 | 22.2±2.3 | 7.92E-27 |
| Salinity (S) | psu | 30.5±3.0 | 32.6±1.0 | 0.000626 |
| Dissolved oxygen (DO) | mg/L | 6.6±0.5 | 7.3±0.3 | 1.10E-7 |
| pH |  | 8.30±0.07 | 8.13±0.07 | 1.06E-13 |
| Suspended solid (SS） | mg/L | 4.9±3.8 | 7.1±7.9 | 0.179117 |
| Chl-*a* | µg/L | 0.63±0.63 | 1.88±1.33 | 0.000018 |

## **Seasonal Variations of Nutrients**

Table 2: Two-tailed independent-sample t-tests between the mean value of nutrients (mean ± SD) in summer and winter in Beibu Gulf, China.

| **Nutrients**  **(**μmol/L) | **Summer** | **Winter** | ***p*** |
| --- | --- | --- | --- |
| SiO_3_^2-^-Si | 35.3±25.0 | 27.8±15.8 | 0.170 |
| PO_4_^3-^-P | 0.18±0.13 | 0.24±0.29 | 0.030 |
| DIN | 3.6±2.4 | 8.1±7.6 | 0.004 |
| NH_4_^+^-N | 1.8±1.8 | 2.6±2.6 | 0.709 |
| NO_3_^-^-N | 1.6±1.6 | 5.0±6.3 | 0.008 |
| NO_2_^-^-N | 0.14±0.25 | 0.45±0.39 | 0.0007 |
| N/P | 77±206 | 96±187 | 0.7 |

## **Dominant Phytoplankton Taxa**

Table 3: The dominant phytoplankton taxa identified in summer and winter based on morphology and metabarcoding identification based on the dominance index (*Y*> 0.02).

| Season | Method | Phytoplankton Taxa | *Y* | Proportion (%) |
| --- | --- | --- | --- | --- |
| Summer | Morphology | *Chaetoceros curvisetus* | 0.242 | 31.51 |
|  |  | *Thalassiosira subtilis* | 0.213 | 24.58 |
|  |  | *Skeletonema costatum* | 0.151 | 18.81 |
|  |  | *Synedra spp.* | 0.052 | 6.77 |
|  |  | *Bacteriastrum hyalinum* | 0.030 | 4.22 |
|  | Metabarcoding | *Heterocapsa circularisquama* | 0.095 | 9.46 |
|  |  | *Scrippsiella trochoidea* | 0.061 | 6.06 |
|  |  | *Dolichomastix tenuilepis* | 0.059 | 5.90 |
|  |  | *Ostreococcus* spp. RCC410 | 0.029 | 2.90 |
|  |  | *Takayama* cf. *pulchellum* | 0.028 | 2.76 |
|  |  | *Gymnodinium* spp. NA-2008 | 0.027 | 2.69 |
| Winter | Morphology | *Phaeocystis globosa* | 0.419 | 59.90 |
|  |  | *Thalassiosira subtilis* | 0.038 | 6.66 |
|  |  | *Thalassionema nitzschioides* | 0.033 | 4.34 |
|  |  | *Trichodesmium erythraeum* | 0.032 | 3.74 |
|  | Metabarcoding | *Micromonas pusilla* | 0.089 | 8.87 |
|  |  | *Stephanodiscus minutulus* | 0.056 | 5.80 |
|  |  | *Phaeocystis* spp. | 0.037 | 3.67 |
|  |  | *Picochlorum* spp. | 0.031 | 3.13 |
|  |  | *Prorocentrum rhathymum* | 0.030 | 3.04 |
|  |  | *Scrippsiella trochoidea* | 0.028 | 2.83 |
|  |  | *Chloropicon maureeniae* | 0.028 | 2.80 |

## **List of Morphospecies Identified**

Table 4: All phytoplankton morphospecies identified in summer and winter based on morphological identification

| **Species/Taxa** | **Summer** | **Winter** | **Bloom Forming** | **Potential Toxicity** |
| --- | --- | --- | --- | --- |
| **Haptophyta** |  |  |  |  |
| *Phaeocystis globosa* |  | + | + | + |
| **Ochrophyta** |  |  |  |  |
| *Dictyocha fibula* | + | + | + |  |
| **Chlorophyta** |  |  |  |  |
| *Chlorella* spp. | + |  |  |  |
| **Bacillariophyta** |  |  |  |  |
| *Detonmula pumila* | + | + |  |  |
| *Chaetoceros curvisetus* | + | + | + |  |
| *Chaetoceros decipiens* | + | + |  |  |
| *Chaetoceros borealis* | + | + |  |  |
| *Chaetoceros danicus* | + |  | + |  |
| *Chaetoceros crinitus* | + |  |  |  |
| *Chaetoceros* spp*.* | + | + |  |  |
| *Chaetoceros coarctatus* | + |  |  |  |
| *Chaetoceros castracanei* | + |  |  |  |
| *Chaetoceros diadema* | + |  | + |  |
| *Chaetoceros pseudocurvisetus* | + | + | + |  |
| *Chaetoceros didymus var.didymus* | + |  | + |  |
| *Chaetoceros costatus* | + |  |  |  |
| *Chaetoceros dsitans* | + | + |  |  |
| *Chaetoceros indicus* | + |  |  |  |
| *Chaetoceros affinis var. affinis* | + |  | + |  |
| *Chaetoceros paradoxus* | + | + |  |  |
| *Chaetoceros rostratus* | + |  |  |  |
| *Chaetoceros siamense* |  | + | + |  |
| *Chaetoceros denticulatus* |  | + |  |  |
| *Chaetoceros vanheurckii* |  | + |  |  |
| *Chaetoceros lorenzianus* |  | + | + |  |
| *Ditylum brightwellii* | + | + | + |  |
| *Ditylum sol* | + | + |  |  |
| *Rhizosolenia bergonii* | + | + |  |  |
| *Rhizosolenia styliformis* | + | + | + |  |
| *Rhizosolenia robusta Norman, 1861* | + | + |  |  |
| *Rhizosolenia setigera* | + | + | + |  |
| *Rhizosolenia* spp*.* | + | + |  |  |
| *Rhizosolenia hyalina* | + |  |  |  |
| *Rhizosolenia alata f.gracillima* | + |  | + |  |
| *Rhizosolenia cochlea* | + | + |  |  |
| *Rhizosolenia sinensis* | + | + |  |  |
| *Rhizosolenia imbricata* |  | + |  |  |
| *Rhizosolenia alata* |  | + | + |  |
| *Guinardia flaccida* | + | + | + |  |
| *Guinardia cylindrus* | + |  | + |  |
| *Bacteriastrum furcatum* | + | + |  |  |
| *Bacteriastrum hyalinum* | + | + |  |  |
| *Nitzschia longissima* | + | + | + |  |
| *Nitzschia lorenziana* | + | + |  |  |
| *Nitzschia* spp. | + | + |  |  |
| *Nitzschia bicapitata* | + |  |  |  |
| *Nitzschia closterium* | + | + | + |  |
| *Eucampia cornuta* | + |  |  |  |
| *Eucampia zodiacus* | + | + | + |  |
| *Odontella aurita* | + | + | + |  |
| *Odontella sinensis* | + | + | + |  |
| *Odontella regia* |  | + |  |  |
| *Leptocylindrus danicus Cleve* | + |  | + |  |
| *Achnanthes brevipes* | + |  |  |  |
| *Thalassiothrix frauenfeldii* | + | + | + |  |
| *Thalassiothrix lineatus* | + | + |  |  |
| *Thalassiosira subtilis* | + | + | + |  |
| *Thalassiothrix longissima* |  | + |  |  |
| *Dactyliosolen blavyanus* | + |  |  |  |
| *Pseudosolenia calcar-avis* | + |  | + |  |
| *Corethron criophilum* | + |  |  |  |
| *Lauderia annulata* | + | + | + |  |
| *Pleurosigma pelagicum* | + | + |  |  |
| *Pleurosigma acutum* |  | + |  |  |
| *Palmeria hardmaniana* | + | + |  |  |
| *Thalassionema nitzschioides* | + | + | + |  |
| *Hemiaulus membranacus* | + |  |  |  |
| *Hemiaulus sinensis* | + | + |  |  |
| *Planktoniella formosa* | + | + |  |  |
| *Skeletonema costatum* | + | + | + |  |
| *Skeletonema tropicum* | + | + |  |  |
| *Gossleriella tropica* | + |  |  |  |
| *Pseudo-nitzschia delicatissima(Cleve) Heiden, 1928* | + |  | + | + |
| *Pseudo-nitzschia pungens* |  | + | + | + |
| *Diatoma hyalinum* | + |  |  |  |
| *Stephanopyxis turris* | + |  | + |  |
| *Stephanopyxis palmeriana* | + | + | + |  |
| *Helicotheca tamesis* | + | + |  |  |
| *Biddulphia puchella* |  | + |  |  |
| *Biddulphia mobiliensis* |  | + | + |  |
| *Coscinodiscus centralis* | + | + | + |  |
| *Coscinodiscus wailesii* | + |  | + |  |
| *Coscinodiscus asteromphalus* | + | + | + |  |
| *Coscinodiscus* spp*.* | + | + |  |  |
| *Coscinodiscus gigas var.gigas* | + |  | + |  |
| *Coscinodiscus radiatus* |  | + | + |  |
| *Coscinodiscus oculusiridis* |  | + |  |  |
| *Coscinodiscus subtilis var. subtilis* |  | + |  |  |
| *Cyclotella* spp. | + | + |  |  |
| *Synedra* spp. | + | + |  |  |
| *Bellerochea horologicalis* | + | + |  |  |
| *Bacillaria paradoxa* |  | + |  |  |
| *Triceratium favus* |  | + |  |  |
| *Navicula* spp. | + | + |  |  |
| *Pinnularia* spp. |  | + |  |  |
| *Paralia sulcata* |  | + |  |  |
| *Gyrosigma normanii* |  | + |  |  |
| *Climacodium frauenfeldianum* |  | + |  |  |
| *Licmophora abbreviata* |  | + |  |  |
| **Cyanophyta** |  |  |  |  |
| *Trichodesmium erythraeum* | + | + | + | + |
| *Trichodesmium hildebrandtii* |  | + | + |  |
| *Trichodesmium thiebautii* | + | + | + |  |
| **Dinophyta** |  |  |  |  |
| *Ceratium macroceros var. macroceros* | + | + |  |  |
| *Ceratium breve var. parallelum* | + |  |  |  |
| *Ceratium fusus var.seta* | + |  | + | + |
| *Ceratium tripos var.pulchellum f.semipulchellum* | + |  | + |  |
| *Ceratium biceps* | + |  |  |  |
| *Ceratium lunula* | + |  |  |  |
| *Ceratium longirostrum* | + |  |  |  |
| *Ceratium furca var.furca* | + | + | + |  |
| *Ceratium euarcuatum* |  | + |  |  |
| *Ceratium inflatum* |  | + |  |  |
| *Dinophysis miles* | + | + |  | + |
| *Protoperidinium venustum* | + | + |  |  |
| *Protoperidinium divergens* |  | + |  |  |
| *Pyrocystis fusiformis f.fusiformis* | + |  |  |  |
| *Pyrocystis fusiformis f.bicornia* |  | + |  |  |
| *Pyrocystis noctiluca* |  | + |  |  |
| *Pyrophacus steinii* | + | + | + |  |

## **Diversity Indexes**

Table 7: Average (mean ± SD) Shannon-Wiener (*H*´), Pielou’s (*J*´) and Margalef (D) diversity indexes based on morphological and metabarcoding approach in summer and winter.

| **Method** | **Season** | **Diversity Index** | **Average** |
| --- | --- | --- | --- |
| Morphology | Summer | *H’* | 1.98±0.49 |
|  |  | *J’* | 0.70±0.17 |
|  |  | D | 0.75±0.22 |
|  | Winter | *H’* | 1.81±0.74 |
|  |  | *J’* | 0.70±0.24 |
|  |  | D | 0.63±0.32 |
| Metabarcoding | Summer | *H’* | 2.25±0.38 |
|  |  | *J’* | 0.45±0.06 |
|  |  | D | 9.27±2.07 |
|  | Winter | *H’* | 2.09±0.60 |
|  |  | *J’* | 0.41±0.11 |
|  |  | D | 9.82±1.82 |


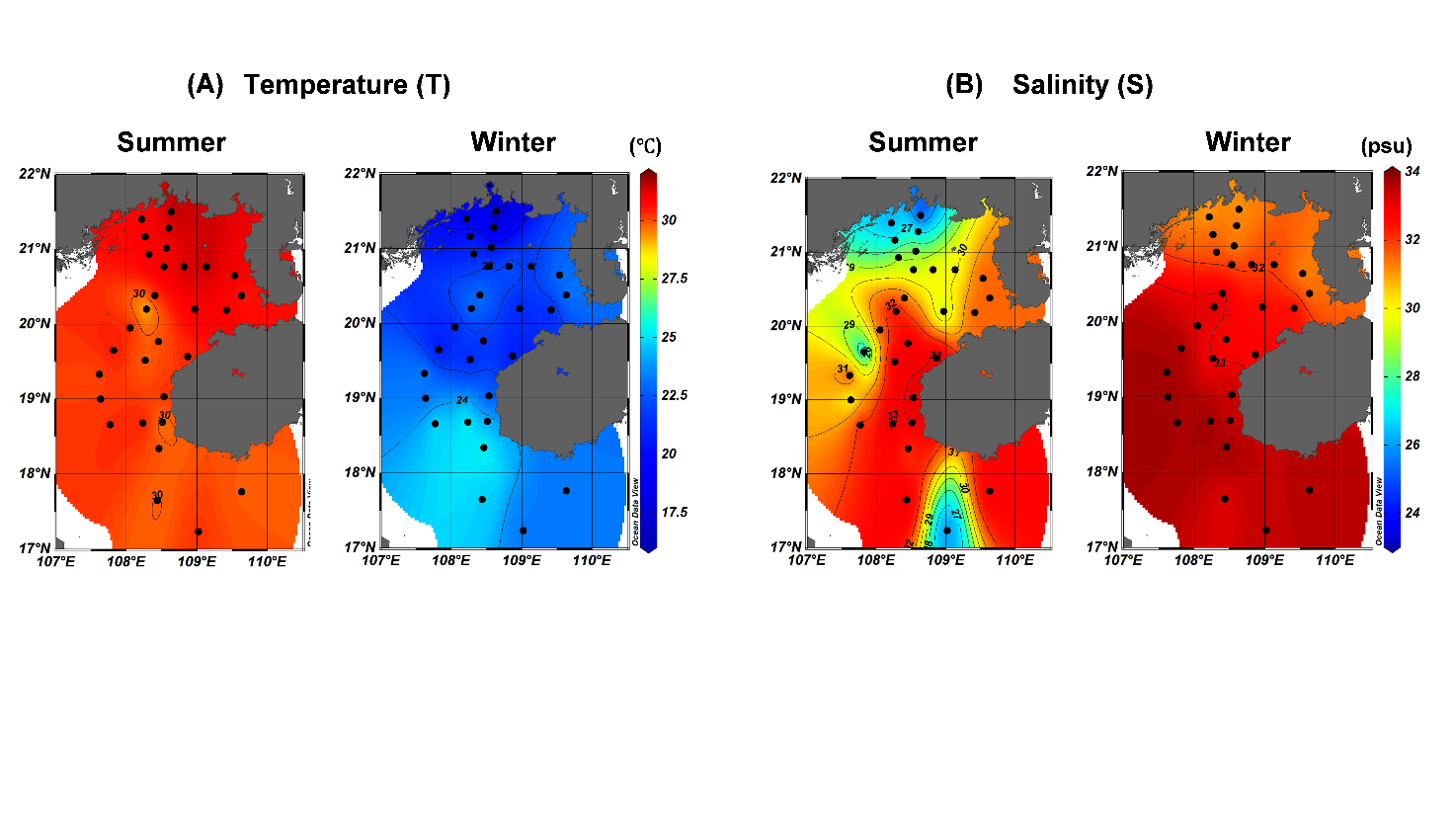


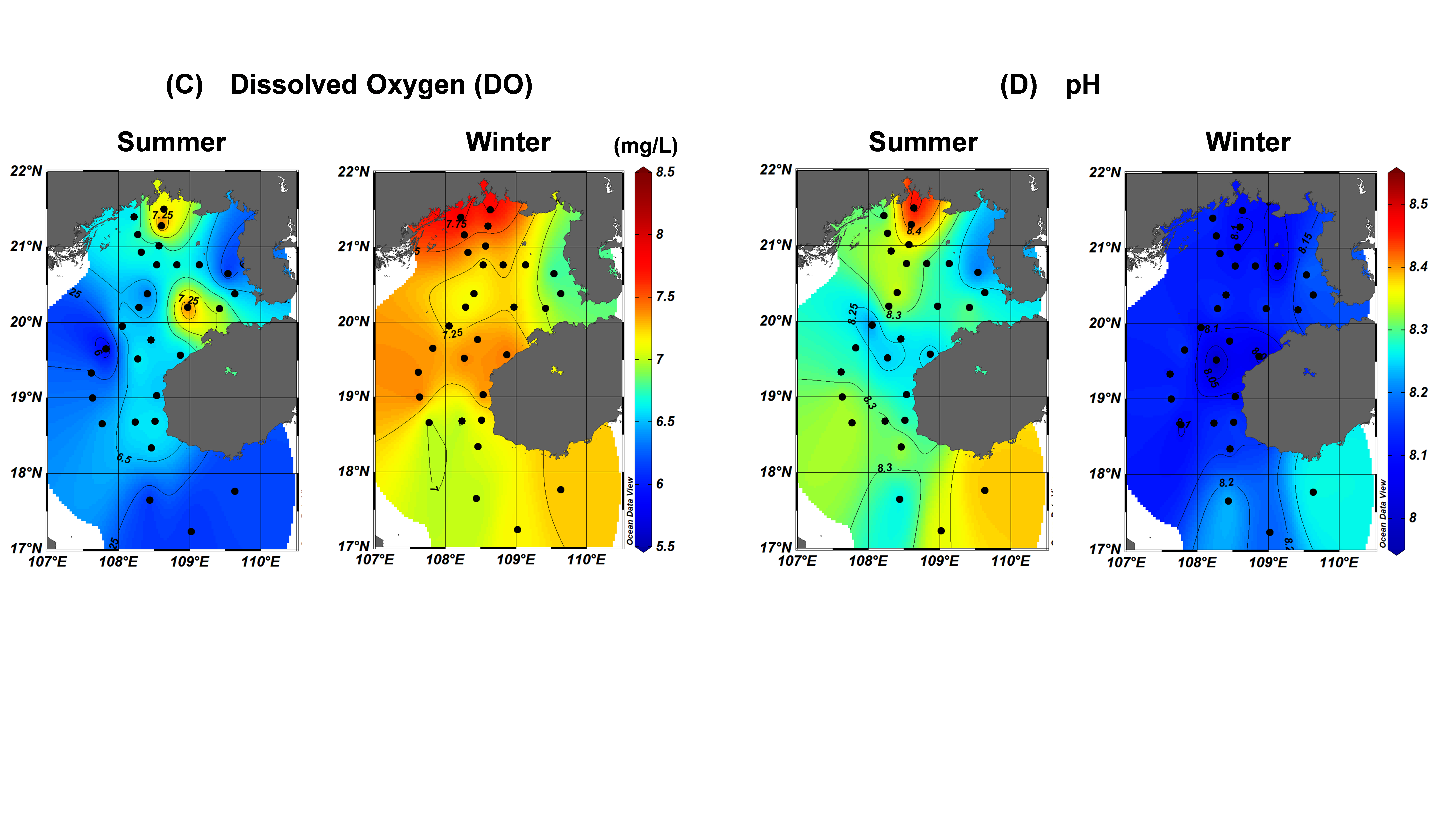


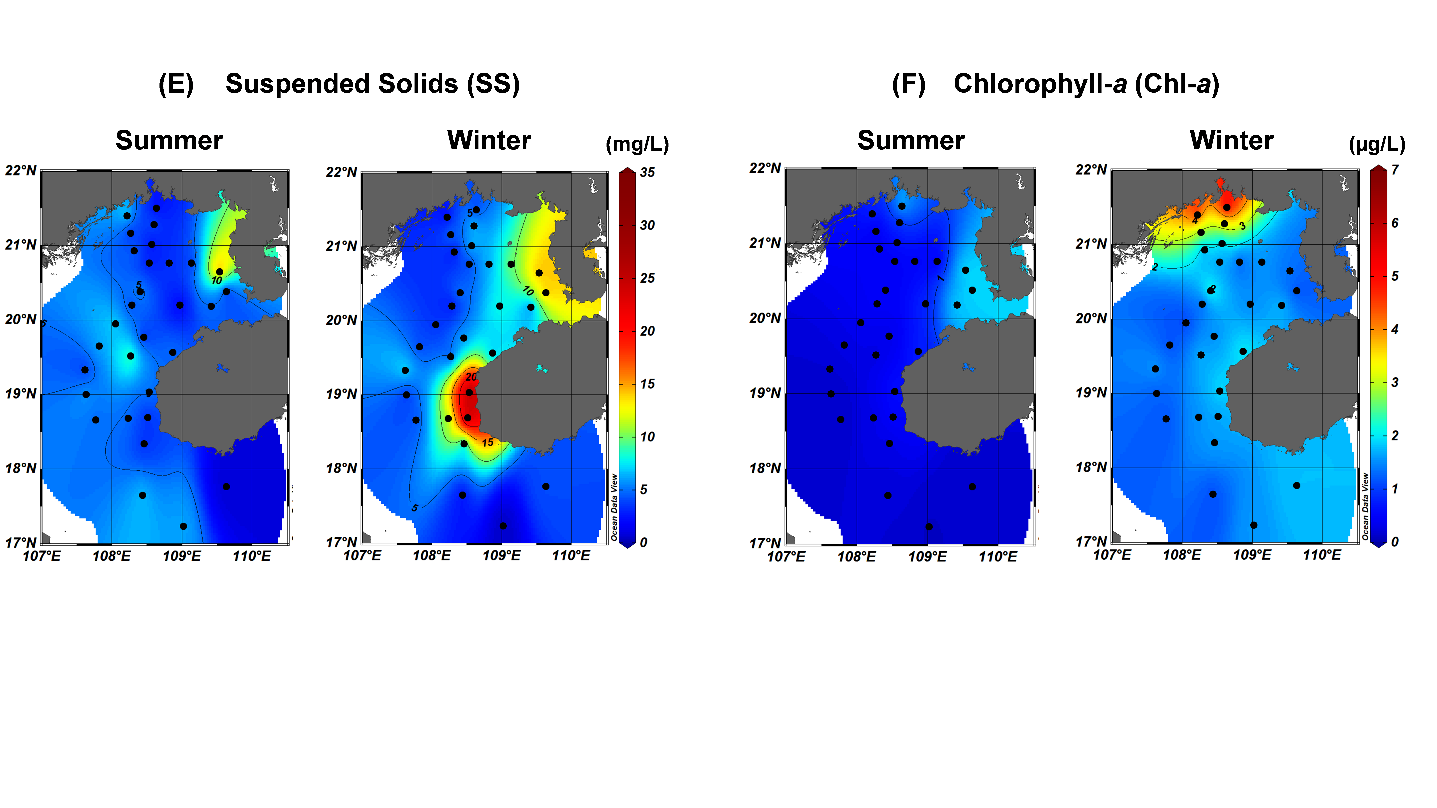


**Fig. S1** Spatial distribution of the sea surface A, temperature (T), B, salinity (S), C, dissolved oxygen (DO), D, pH, E, suspended solids (SS) and F, Chlorophyll-a (Chl-*a*), in summer and winter

**
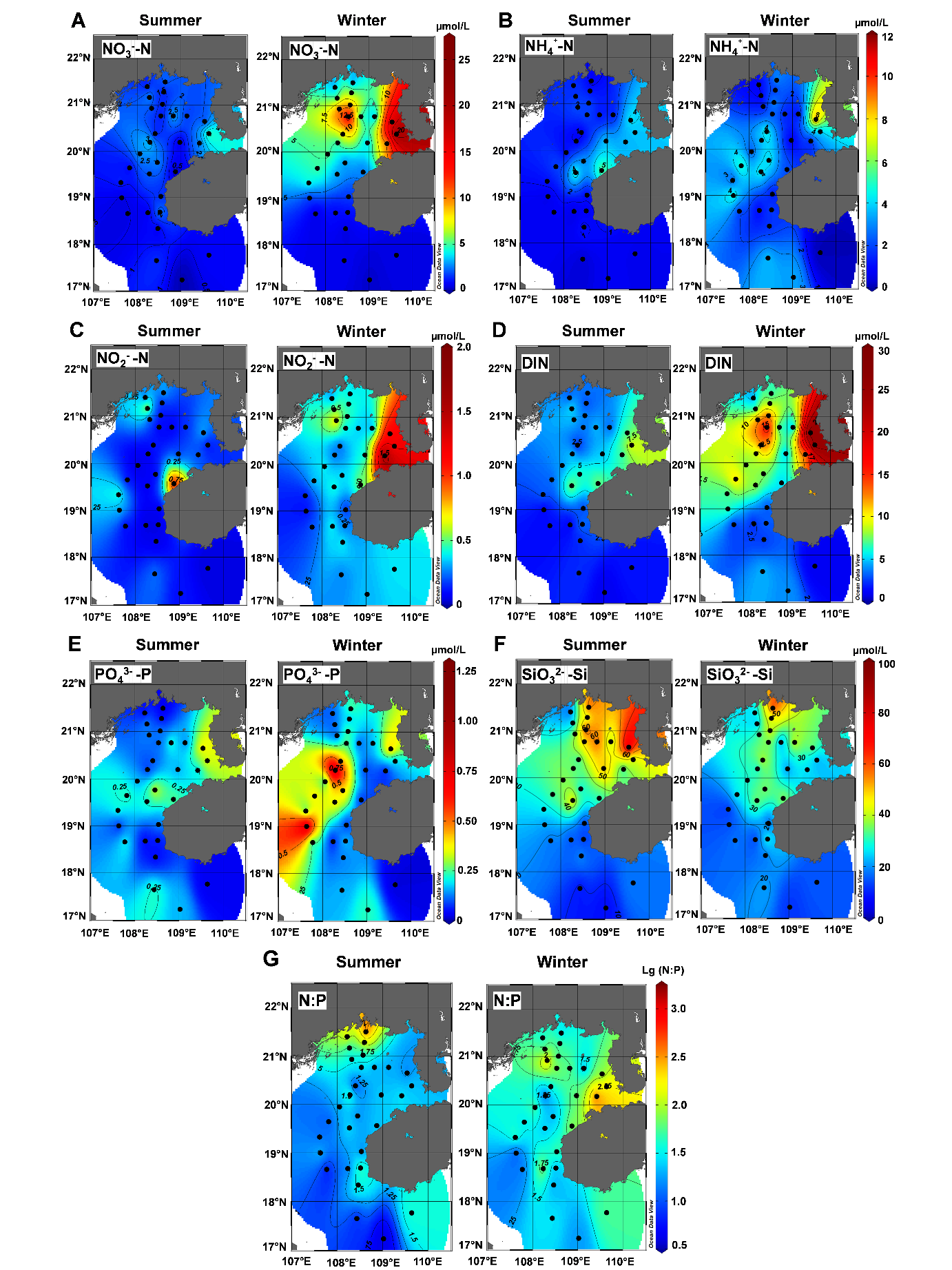
**

**Fig. S2** Nutrient distribution heatmap of: A, nitrate (NO_3_^-^-N), B, ammonium (NH_4_^+^-N), C, nitrite (NO_2_^-^-N), D, dissolved inorganic nitrogen (DIN), E, phosphate (PO_4_^3-^-P), F, silicate (SiO_3_^2-^-Si) and G, N:P ratio in summer and winter at Beibu Gulf.


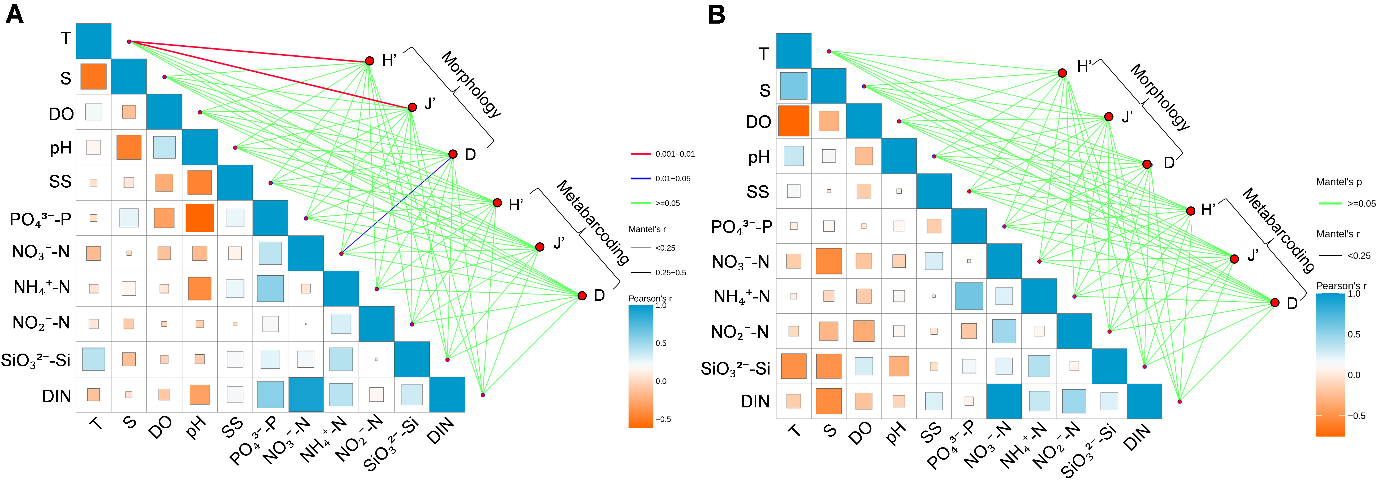


**Fig. S3** Mantel’s test between the Shannon-Wiener index (H’), Pielou’s index (J’) and Margalef index (D) with temperature (T), salinity (S), dissolved oxygen (DO), pH, suspended solids (SS), NO_3_^-^-N, NO_2_^-^-N, NH_4_^+^-N, DIN, SiO_3_^2-^-Si and PO_4_^3-^-P.
